# CT-assessed abdominal visceral adiposity and MASLD: a sex-stratified cross-sectional analysis

**DOI:** 10.3389/fnut.2026.1750470

**Published:** 2026-03-25

**Authors:** Linying Xu, Ting Chen, Yitian Xie, Xueyu Xiang, Yupeng Liu, Chongyong Xu, Shuran Wang

**Affiliations:** 1 Department of Nutrition and Food Hygiene, School of Public Health, Wenzhou Medical University, Wenzhou, China; 2 Department of Epidemiology and Biostatistics, School of Public Health, Wenzhou Medical University, Wenzhou, China; 3 Department of Radiology, The Second Affiliated Hospital and Yuying Children's Hospital of Wenzhou Medical University, Wenzhou, China

**Keywords:** adipose tissue, body composition, computerized tomography, metabolic dysfunction-associated steatotic liver disease, obesity

## Abstract

**Aims:**

To test the hypothesis that CT-derived visceral fat and muscle measures are more strongly associated with MASLD than BMI, and that these associations are sex-specific and nonlinear, we examined abdominal body composition in relation to MASLD risk.

**Methods:**

In this cross-sectional study of 7,805 Chinese adults who underwent non-contrast abdominal CT between September 2020 and June 2023, body composition was quantified, including visceral fat area (VFA), subcutaneous fat area (SFA), skeletal muscle area (SMA), L3 skeletal muscle index (L3SMI), and CT-derived waist circumference(CT-WC). Participants were drawn from hospitalized patients undergoing CT for clinical indications (*n* = 7,051) and individuals attending routine health examinations (*n* = 754). MASLD was defined according to the 2023 criteria. Associations with MASLD were assessed using logistic regression, spline modeling, and ROC analyses.

**Results:**

Among 7,805 participants (mean age 47.5 years; 62.2% men), MASLD prevalence was 25.8% (28.1% in men and 22.0% in women). In sex-specific fully adjusted models, VFA showed the strongest and most consistent association with MASLD. Men in the highest VFA quartile had an odds ratio (OR) of 11.5 (95% CI 8.3–16.1) compared with the lowest quartile, while women exhibited a markedly steeper risk gradient (OR 32.5, 95% CI 17.3–67.0). Waist circumference (WC) showed similar independent associations, whereas BMI was not independently associated with MASLD in men but remained significant in women. Subcutaneous fat and skeletal muscle measures were weakly associated or not significant after adjustment. Restricted cubic spline analyses revealed pronounced nonlinear and threshold effects for visceral fat, with risk rising sharply beyond sex-specific VFA thresholds. CT-derived body composition models improved MASLD discrimination compared with BMI alone (AUC 0.76 vs. 0.70 in men; 0.83 vs. 0.77 in women). Associations were consistent across metabolic subgroups and robust to sensitivity analyses, including E-value assessment for unmeasured confounding.

**Conclusion:**

CT-based measures of visceral fat may provide significant incremental value over BMI in MASLD risk assessment. Findings suggest that MASLD risk may differ by sex and appears to be more closely related to fat distribution than to total adiposity, highlighting the potential need to incorporate central obesity assessment into clinical and public health strategies.

## Introduction

Metabolic dysfunction-associated fatty liver disease (MASLD), formerly termed non-alcoholic fatty liver disease (NAFLD), has emerged as a global health challenge (1). Currently, the prevalence of MASLD among adults is approximately 38%, while in children and adolescents it ranges from 7 to 14%. By 2040, the prevalence of MASLD in adults is projected to exceed 55% (2). The burden of MASLD is becoming increasingly severe, affects more than one-third of adults worldwide and is increasingly recognised as part of the spectrum of cardiometabolic disorders. MASLD may progress to fibrosis, cirrhosis and hepatocellular carcinoma and is a leading contributor to cardiovascular morbidity and mortality (3). Despite its high prevalence, the heterogeneity of MASLD risk among individuals with similar body weight underscores the limitations of conventional obesity indices (4).

Body mass index(BMI), the most commonly used obesity index, fails to distinguish fat from lean mass and cannot capture regional fat distribution (5, 6). Emerging evidence highlights that the health consequences of adiposity depend on its location: visceral adipose tissue (VAT), located within the peritoneal cavity and drained via the portal vein, releases free fatty acids and inflammatory mediators that promote insulin resistance and hepatocellular lipid accumulation (7–9). In contrast, subcutaneous adipose tissue (SAT) acts as a safer energy reservoir and may limit ectopic fat deposition. Skeletal muscle also influences glucose homeostasis through insulin-mediated glucose disposal; reduced muscle mass may therefore exacerbate metabolic dysfunction (10). However, metabolic risk is not determined by muscle mass alone and may also be influenced by muscle quality and composition, such as intramuscular fat infiltration. CT-based measurements at the third lumbar vertebra (L3) enable accurate quantification of skeletal muscle and adipose tissue compartments, with L3 muscle area strongly correlating with total body muscle mass (11, 12). Although CT can also capture aspects of muscle quality and density, the present study focused on muscle quantity-based measures.

Previous studies linking CT-derived body composition to NAFLD have largely been conducted in non-Asian populations and rarely included systematic sex-stratified analyses. The nonlinear associations between fat distribution and MASLD, as well as potential differences between men and women, remain incompletely characterized. Notably, the association between VAT and metabolic disease risk is often non-linear and may involve threshold effects (13); identifying such inflection points is therefore important for improving risk stratification and informing both public health strategies and clinical diagnostic cutoffs. We hypothesised that visceral adiposity measured by CT would be a stronger predictor of MASLD than BMI and that the association would differ by sex. To test this hypothesis, we analysed a large cohort of patients who underwent abdominal CT for diverse clinical indications, quantified abdominal fat and muscle compartments, and explored their sex-specific and nonlinear relationships with MASLD.

## Methods

### Study design and population

This retrospective cross-sectional study used de-identified electronic medical records from the Second Affiliated Hospital of Wenzhou Medical University in Wenzhou, China. The study protocol was reviewed and approved by the institutional ethics committee (Approval No. 2022-K-306-1), with a waiver of informed consent due to its retrospective and anonymized nature of the data.

Adults aged 18–65 years who underwent non-contrast abdominal computed tomography (CT) scans between September 2020 and June 2023 were eligible for inclusion. The upper age limit was prespecified to reduce heterogeneity related to advanced age, including age-related sarcopenia, multimorbidity, and medication burden, and to focus on adults aged 18–65 years in whom early identification and prevention of MASLD are particularly relevant.

Participants were predominantly hospital-based inpatients who underwent abdominal CT for routine clinical indications, supplemented by a smaller cohort of individuals undergoing routine health examinations at the same institution. CT scans were not performed for the purpose of assessing hepatic steatosis or body composition.

To minimise confounding, we excluded participants with: (1) known malignancy, sepsis, cirrhosis, viral hepatitis, or autoimmune liver disease; (2) significant alcohol consumption (> 20 g/day in women or > 30 g/day in men); (3) incomplete clinical, laboratory, or imaging data; (4) poor-quality CT images (e.g., motion artefacts, metallic implants, or inappropriate phase); or (5) pregnancy.

**Figure 1:**
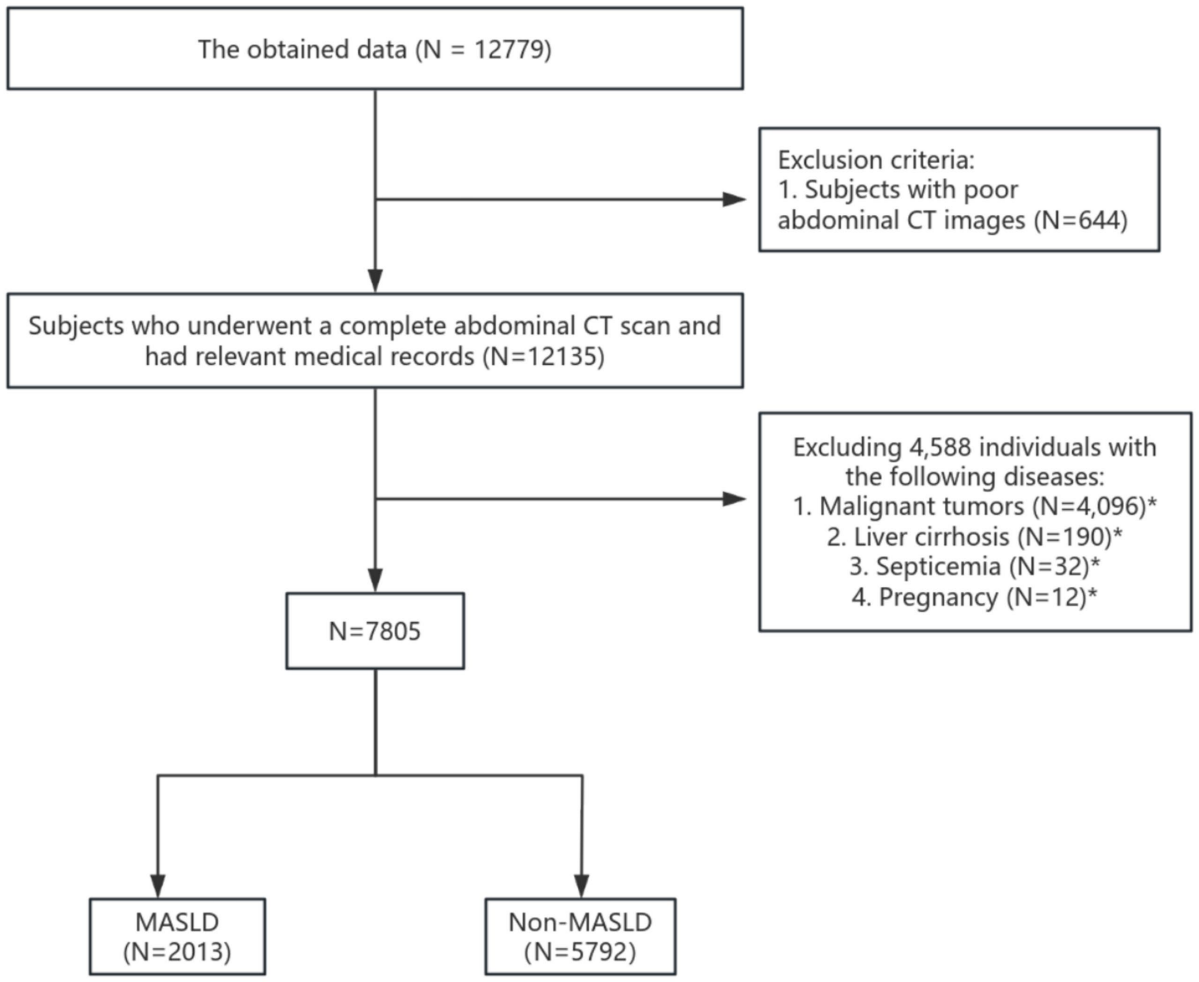
Research flowchart. Among the subjects with poor abdominal CT images, including ① space-occupying lesions (*n* = 129), ② effusion (*n* = 133), ③ pneumoperitoneum (*n* = 134), ④ intestinal problems such as intestinal obstruction, intestinal distension, intestinal wall thickening, and intestinal dilation (*n* = 163), ⑤ others (*n* = 76). *Indicates that there may be overlaps in these data, but the data shown in the graph do not have any overlaps.

A total of 12,779 individuals with abdominal CT scans were initially screened. After applying the exclusion criteria, 7,805 participants were included in the final analytical cohort. The participant selection process is illustrated in Figure 1.

The study population comprised 7,805 participants recruited between September 2020 and June 2023 from two data sources within the same institution: a hospital-based inpatient cohort (*n* = 7,051) undergoing abdominal CT for clinical indications, and a health examination cohort (*n* = 754) consisting of asymptomatic or subclinical individuals who voluntarily underwent abdominal CT as part of routine health assessments. This mixed-cohort design was intended to capture a broader range of health status and body composition profiles.

### Data collection

Demographics characteristics (age and sex), anthropometric measures (height and weight), comorbidities (type 2 diabetes, hypertension, and dyslipidaemia), and lifestyle factors (smoking status and alcohol consumption) were extracted from electronic medical records. Body mass index (BMI) was calculated as weight (kg) divided by height squared (m^2^).

Laboratory data were obtained from the record closest to the CT examination date, within a maximum window of 6 months. This window was selected to balance temporal proximity with data completeness in this cross-sectional study. These included alanine aminotransferase (ALT), aspartate aminotransferase (AST), albumin, direct bilirubin, fasting plasma glucose, glycated haemoglobin, total cholesterol, low-density lipoprotein cholesterol, high-density lipoprotein cholesterol, triglycerides and uric acid. Missing covariate values (< 10% for any variable) were imputed using multivariate imputation by chained equations with five imputed datasets.

### CT acquisition and body-composition assessment

**Figure 2:**
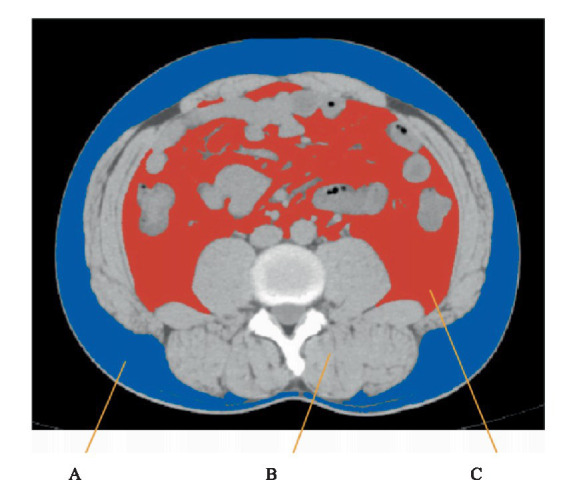
This image selects the abdominal CT scan at the lower edge of L3. A, Abdominal subcutaneous adipose; B, abdominal muscles; C, abdominal visceral fat.

All CT scans were performed using a standardized protocol on Philips Brilliance 16-slice scanner (120 kV, 200 mA, 5 mm slice thickness), with participants in the supine position during a single breath-hold. A single axial image at the level of the third lumbar vertebra (L3) was selected for body-composition analysis, as this level has been shown to correlate strongly with whole-body fat and muscle mass (see Figure 2).

CT-derived body composition metrics were the primary exposures of interest and were defined *a priori*. Image analysis was conducted using ImageJ software (version 1.53c, USA). Tissue compartments were segmented semi-automatically using validated Hounsfield unit (HU) thresholds: −150 to −50 HU for VFA, −190 to −30 HU for SFA, and −29 to +150 HU for SMA (14–16). SMA included psoas, paraspinal, and abdominal wall muscles, and L3SMI was calculated as SMA/height^2^ (cm^2^/m^2^). CT-WC was measured by tracing the body contour at the L3 level using a − 250 to +1,000 HU threshold. All measurements were performed by trained analysts blinded to clinical data.

### Definition of MASLD

The primary outcome was MASLD, defined according to the 2023 multisociety consensus as imaging-confirmed hepatic steatosis plus at least one metabolic abnormality. Hepatic steatosis was identified based on unenhanced CT (e.g., liver attenuation ≤40 HU or liver-to-spleen attenuation ratio <1.0) and/or documented ultrasound reports indicating fatty liver in the medical record. Metabolic abnormalities included: (1) adiposity or central obesity (BMI ≥ 23 kg/m^2^, or elevated WC); (2) dysglycemia or diabetes (FPG ≥ 100 mg/dL, HbA1c elevation, type 2 diabetes, or glucose-lowering therapy); (3) hypertension (blood pressure ≥ 130/85 mmHg or antihypertensive therapy); (4) hypertriglyceridemia (triglycerides ≥ 150 mg/dL or lipid-lowering therapy); or (5) low high-density lipoprotein cholesterol (HDL) (< 40 mg/dL in men or < 50 mg/dL in women).

### Statistical analysis

All analyses were conducted using R software (version 4.3.0) and MedCalc (version 23.2.1). Continuous variables are presented as mean ± standard deviation (SD) or median (interquartile range), and categorical variables as number (percentage). Group comparisons were performed using Student’s t tests or Mann–Whitney U tests for continuous variables and Chi-square test for categorical variables. A two-tailed *p* < 0.05 denoted statistical significance. This study was reported in accordance with the STROBE guideline.

Given established biological differences in fat distribution and MASLD risk between sexes, all analyses were prespecified and conducted separately in men and women. Logistic regression models were used to estimate odds ratios (ORs) and 95% confidence intervals (CIs) for MASLD in relation to each body-composition metric (BMI, VFA, SFA, WC, SMA, L3SMI). Exposures were analyzed both continuously (per standard deviation increase) and categorically (sex-specific quartiles), with trends assessed across quartiles.

To evaluate the independent contribution of specific fat depots beyond general obesity, three sequential models were constructed: Model 1 (unadjusted), Model 2 (adjusted for age), and Model 3 (DAG-informed fully adjusted using exposure-specific covariate sets, including mutual adjustment for BMI and central obesity measures where appropriate, Supplementary Table S1). This strategy accounts for differing confounding structures across body composition metrics and avoids overadjustment or adjusting for mediators.

Restricted cubic spline (RCS) models with 3–5 knots were used to explore potential non-linear and threshold associations between body composition measures and MASLD risk, with knot selection guided by Bayesian information criteria and clinical interpretability. Non-linearity was assessed using likelihood ratio tests comparing spline and linear terms.

To assess discriminatory performance, receiver operating characteristic (ROC) curve analyses were performed comparing models including BMI alone, BMI plus CT-derived fat distribution measures, and BMI plus fat and muscle measures. Sensitivity analyses including subgroup analyses stratified by age and metabolic comorbidities and E-value anslyses were conducted to assess consistency of associations and the robustness of the main findings. The results, together with the calculation method, are provided in Supplementary Tables S15, S16.

As a sensitivity analysis, propensity score matching (PSM) was performed to further balance baseline covariates between participants with and without MASLD. Details of the matching procedure and results are provided in the Supplementary materials.

## Results

### Baseline characteristics

A total of 7,805 participants were included in the final analysis, with a mean age of 47.5 ± 11.0 years. Of these, 4,858 (62.2%) were men and 2,947 (37.8%) were women. Overall, 2,013 participants met the diagnostic criteria for MASLD, yielding a prevalence of 25.8%. MASLD prevalence was higher in men than in women (28.1% vs. 22.0%, respectively). The study population included both hospitalized patients (*n* = 7,051) and individuals undergoing routine health examinations (*n* = 754). Accordingly, Table 1 presents the baseline characteristics of the overall study cohort, while detailed characteristics stratified by data source are available from the corresponding author upon request.

**Table 1:** Basic characteristics of the population covered in this study.

Characteristics		Total (*n* = 7,805)	Male (*n* = 4,858)			Female (*n* = 2,947)		
			Non-MASLD (*n* = 3,489)	MASLD (*n* = 1,369)	*p*-value	Non-MASLD (*n* = 2,303)	MASLD (*n* = 644)	*p*-value
Age (years)		47.46 (11.67)	47.75 (11.70)	46.93 (11.09)	0.0254	46.40 (12.00)	50.77 (10.79)	<0.0001
Height (cm)		165.26 (7.67)	169.15 (5.80)	170.00 (5.67)	<0.0001	158.51 (5.17)	158.30 (5.13)	0.3587
Weight (kg)		65.22 (11.63)	67.44 (10.23)	74.78 (10.03)	<0.0001	56.35 (8.67)	64.57 (9.38)	<0.0001
BMI (kg/m^2^)		23.79 (3.40)	23.54 (3.14)	25.85 (3.01)	<0.0001	22.42(3.19)	25.74 (3.33)	<0.0001
SBP (mmHg)		130.09 (20.48)	131.08 (20.37)	136.27 (19.77)	<0.0001	124.40(19.71)	131.98 (20.23)	<0.0001
DBP (mmHg)		81.73 (13.31)	82.26 (13.35)	86.68 (13.47)	<0.0001	77.88 (12.25)	82.11 (12.34)	<0.0001
Blood biochemical indicators								
Prealbumin (mg/L)		244.43 (76.97)	251.75 (79.51)	269.46 (80.59)	<0.0001	219.08 (64.09)	242.28 (71.89)	<0.0001
Alanine Aminotransferase (U/L)		24.28 (13.13)	24.31 (12.56)	31.69 (13.82)	<0.0001	19.18 (11.14)	26.68 (13.05)	<0.0001
Aspartate Aminotransferase (U/L)		23.44 (8.49)	23.64 (8.45)	25.76 (8.85)	<0.0001	21.54 (7.95)	24.21 (8.38)	<0.0001
Alkaline Phosphatase (U/L)		77.06 (22.26)	77.95 (22.00)	79.38 (21.44)	0.0402	73.13 (22.44)	81.31 (22.72)	<0.0001
Gamma-Glutamyl Transferase (U/L)		35.00 (22.84)	36.45 (22.23)	47.22 (23.58)	<0.0001	24.80 (18.48)	37.64 (23.20)	<0.0001
Total Protein (g/L)		67.21 (7.44)	66.284(7.60)	67.63 (7.11)	<0.0001	67.72 (7.27)	69.54 (7.14)	<0.0001
Albumin (g/L)		151.46 (115.10)	155.19 (117.76)	169.74 (127.26)	0.0002	134.25 (100.14)	153.89 (115.57)	<0.0001
Globulin (g/L)		25.31 (4.11)	24.61 (4.05)	25.00 (3.87)	0.0022	26.094 (4.081)	26.959 (4.050)	<0.0001
Total Bilirubin (μmol/L)		13.60 (5.77)	14.27 (5.90)	14.602 (5.98)	0.0813	12.31 (5.28)	12.46 (5.24)	0.5071
Direct Bilirubin (μmol/L)		3.53 (1.65)	3.82 (1.69)	3.70 (1.70)	0.0375	3.14 (1.51)	3.05 (1.47)	0.1923
Unconjugated Bilirubin (μmol/L)		9.94 (4.26)	10.31 (4.37)	10.72 (4.43)	0.0032	9.08 (3.87)	9.29 (3.94)	0.2259
Cholinesterase (U/L)		8347.22 (2133.39)	8160.61 (2140.03)	9178.02 (2147.70)	<0.0001	7898.81 (1934.41)	9195.72 (2021.48)	<0.0001
Fasting Blood Glucose (mmol/L)		5.62 (1.10)	5.62 (1.11)	5.85 (1.14)	<0.0001	5.43 (1.05)	5.83 (1.08)	<0.0001
Glucose (mmol/L)		6.86 (1.84)	6.85 (1.80)	7.13 (2.05)	<0.0001	6.63(1.69)	7.10 (2.02)	<0.0001
Hemoglobin A1c (%)		5.56 (0.48)	5.54 (0.48)	5.71 (0.50)	<0.0001	5.45 (0.43)	5.72 (0.48)	<0.0001
Triglycerides (mmol/L)		1.49 (0.66)	1.49 (0.65)	1.87 (0.67)	<0.0001	1.22 (0.56)	1.70 (0.64)	<0.0001
Total Cholesterol (mmol/L)		4.42 (1.00)	4.29(0.99)	4.57 (1.01)	<0.0001	4.45 (0.97)	4.75 (0.98)	<0.0001
HDL-C (mmol/L)		1.08 (0.31)	1.04 (0.30)	0.95 (0.27)	<0.0001	1.21 (0.32)	1.12 (0.28)	<0.0001
LDL-C (mmol/L)		2.77 (0.87)	2.69 (0.86)	2.86 (0.92)	<0.0001	2.76 (0.81)	3.03 (0.88)	<0.0001
CT measurement indicators								
VFA (mm^2^)		11219.63 (6742.74)	11137.26 (6707.54)	17130.34 (6346.51)	<0.0001	7321.72 (4372.29)	13040.26 (4631.28)	<0.0001
SFA (mm^2^)		14185.99 (6498.43)	11639.35 (5248.10)	15638.94 (6461.38)	<0.0001	15424.55 (6250.26)	20465.02 (7055.44)	<0.0001
Waist circumference (mm)		886.12 (106.30)	883.33 (97.91)	968.60 (92.72)	<0.0001	829.64 (93.90)	927.94 (87.12)	<0.0001
FERET (mm)		308.00 (32.22)	305.90 (29.13)	329.03 (28.45)	<0.0001	294.31 (31.34)	323.65 (29.62)	<0.0001
MINIFERET (mm)		208.66 (32.60)	209.45 (29.64)	236.45 (28.36)	<0.0001	188.30 (26.68)	218.13 (25.54)	<0.0001
SMA (mm^2^)		14843.21 (3616.60)	16416.62 (2496.07)	17771.10 (2794.00)	<0.0001	11443.25 (2654.97)	12253.51 (1817.14)	<0.0001
Calculate indicators								
L3SMI (cm^2^/m^2^)		53.90 (10.96)	57.36 (8.129)	61.45 (8.95)	<0.0001	45.568 (10.86)	48.899 (6.86)	<0.0001
Hyperlipidemia (*n*, %)	Yes	4,031 (51.60)	1949 (55.86)	989 (72.24)		756 (32.83)	337 (52.33)	<0.0001
	No	3,774(48.40)	1,540 (44.14)	380 (27.76)	<0.0001	1,547 (67.17)	307 (47.67)	
Diabetes (*n*, %)	Yes	1965 (25.20)	876 (25.11)	467 (34.11)		415 (18.02)	207 (32.14)	<0.0001
	No	5,840(74.80)	2,613 (74.89)	902 (65.89)	<0.0001	1888 (81.98)	437 (67.86)	
Hypertension (*n*, %)	Yes	3,592 (46.00)	1,641 (47.03)	828 (60.48)		763 (33.13)	360 (55.90)	<0.0001
	No	4,213(54.00)	1848 (52.97)	541 (39.52)	<0.0001	1,540 (66.87)	284 (44.10)	
Smoking (*n*, %)	Yes	1,258 (16.10)	882 (25.28)	368 (26.88)		6 (0.26)	2 (0.31)	1.000
	No		2,607 (74.72)	1,001 (73.12)	0.266	2,297 (99.74)	642 (99.69)	
Drinking (*n*, %)	Yes	960 (12.30)	693 (19.86)	226 (16.51)		36 (1.56)	5 (0.78)	0.188
	No		2,796 (80.14)	1,143 (83.49)	0.0082	2,267 (98.44)	639 (99.22)	

MASLD, metabolic dysfunction-associated steatotic liver disease; BMI body mass index; SBP systolic blood pressure; DBP diastolic blood pressure; HDL-C, high-density lipoprotein; LDL-C, low-density lipoprotein; VFA, visceral fat area; SFA, subcutaneous fat; SMA, skeletal muscle area; L3SMI,the third lumbar skeletal muscle index. Model 3 covariates were exposure-specific, following DAG-based adjustment sets (see methods and Supplementary Table S1).

Baseline characteristics stratified by sex and MASLD status are presented in Table 1. Across both sexes, participants with MASLD exhibited a less favorable cardiometabolic profile compared with those without MASLD, including higher blood pressure, adverse lipid and glycemic parameters, and markedly greater CT-derived measures of central adiposity. Notably, these patterns were consistent in both men and women, highlighting sex-specific differences in fat distribution and cardiometabolic risk. In contrast, differences in skeletal muscle indices were less pronounced after accounting for adiposity measures, suggesting that fat distribution rather than overall body size was a key differentiator between MASLD and non-MASLD groups.

### Associations between CT-derived body composition measures and MASLD

We next evaluated associations between CT-derived body composition measures and the odds of MASLD using sex-stratified logistic regression models (Tables 2, 3). Model 1 was unadjusted, Model 2 adjusted for age, and Model 3 was fully adjusted using exposure-specific, DAG-informed covariate sets (see methods and Supplementary Table S1).

**Table 2:** The logistic regression model based on CT measurements of obesity indicators was used to predict the occurrence of MASLD in males.

Variable		Model 1		Model 2		Model 3	
		OR (95% CI)	*p*-value	OR (95% CI)	*p*-value	OR (95% CI)	*p*-value
BMI	Q1	1 (reference)		1 (reference)		1 (reference)	
	Q2	3.14 (2.47, 4.02)	<0.0001	3.21 (2.52, 4.11)	<0.0001	1.73 (1.34, 2.26)	<0.0001
	Q3	6.02 (4.78, 7.63)	<0.0001	6.14 (4.88, 7.79)	<0.0001	2.18 (1.66, 2.87)	<0.0001
	Q4	9.22 (7.34, 11.67)	<0.0001	9.29 (7.40, 11.76)	<0.0001	1.77 (1.29, 2.43)	0.0004
	*P* for trend		<0.0001		<0.0001		0.0023
	Per SD increment	2.16 (2.01, 2.33)	<0.0001	2.16 (2.01, 2.32)	<0.0001	1.06 (0.93, 1.20)	0.3670
VFA	Q1	1 (reference)		1 (reference)		1 (reference)	
	Q2	4.62 (3.48, 6.20)	<0.0001	5.00 (3.77, 6.34)	<0.0001	3.70 (2.75, 5.05)	<0.0001
	Q3	10.44 (7.97, 13.90)	<0.0001	11.54 (8.78, 15.41)	<0.0001	7.49 (5.53, 10.26)	<0.0001
	Q4	18.19 (13.91, 24.17)	<0.0001	20.59 (15.67, 27.48)	<0.0001	11.51 (8.31, 16.13)	<0.0001
	*P* for trend		<0.0001		<0.0001		<0.0001
	Per SD increment	2.51 (2.33, 2.71)	<0.0001	2.60 (2.41, 2.81)	<0.0001	2.12 (1.92, 2.35)	<0.0001
SFA	Q1	1 (reference)		1 (reference)		1 (reference)	
	Q2	3.17 (2.53, 4.00)	<0.0001	3.18 (2.54, 4.01)	<0.0001	1.61 (1.26, 2.07)	0.0002
	Q3	4.05 (3.25, 5.09)	<0.0001	4.06 (3.25, 4.10)	<0.0001	1.52 (1.18, 1.97)	0.0015
	Q4	8.09 (6.51, 10.11)	<0.0001	8.06 (6.49, 10.09)	<0.0001	2.05 (1.55, 2.72)	<0.0001
	*P* for trend		<0.0001		<0.0001		<0.0001
	Per SD increment	2.05 (1.91, 2.21)	<0.0001	2.08 (1.93, 2.24)	<0.0001	1.29 (1.17, 1.42)	<0.0001
CT-WC	Q1	1 (reference)		1 (reference)		1 (reference)	
	Q2	3.26 (2.52, 4.25)	<0.0001	3.37 (2.60, 4.40)	<0.0001	2.48 (1.88, 3.31)	<0.0001
	Q3	7.53 (5.90, 9.72)	<0.0001	7.79 (6.09, 10.06)	<0.0001	4.77 (3.55, 6.48)	<0.0001
	Q4	13.23 (10.38, 17.04)	<0.0001	13.46 (10.56, 17.35)	<0.0001	6.24 (4.36, 8.99)	<0.0001
	*P* for trend		<0.0001		<0.0001		<0.0001
	Per SD increment	2.59 (2.40, 2.81)	<0.0001	2.59 (2.39, 2.81)	<0.0001	2.28 (1.96, 2.65)	<0.0001
SMA	Q1	1 (reference)		1 (reference)		1 (reference)	
	Q2	1.50 (1.23, 1.83)	<0.0001	1.50 (1.23, 1.83)	<0.0001	1.09 (0.88, 1.36)	0.4363
	Q3	2.00 (1.65, 2.44)	<0.0001	2.02 (1.66, 2.46)	<0.0001	1.12 (0.90, 1.40)	0.3013
	Q4	3.74 (3.10, 4.52)	<0.0001	3.79 (3.13, 4.59)	<0.0001	1.45 (1.14, 1.84)	0.0022
	*P* for trend		<0.0001		<0.0001		0.0020
	Per SD increment	1.69 (1.58, 1.81)	<0.0001	1.71 (1.59, 1.83)	<0.0001	1.17 (1.07, 1.27)	0.0005
L3SMI	Q1	1 (reference)		1 (reference)		1 (reference)	
	Q2	1.49 (1.22, 1.82)	0.0001	1.49 (1.22, 1.82)	0.000106	1.10 (0.89, 1.37)	0.3804
	Q3	2.12 (1.75, 2.57)	<0.0001	2.11 (1.74, 2.56)	<0.0001	1.28 (1.03, 1.60)	0.0274
	Q4	3.52 (2.92, 4.26)	<0.0001	3.50 (2.90, 4.23)	<0.0001	1.53 (1.20, 1.95)	0.0006
	*P* for trend		<0.0001		<0.0001		0.0002
	Per SD increment	1.63 (1.53, 1.74)	<0.0001	1.63 (1.52, 1.74)	<0.0001	1.18 (1.08, 1.29)	0.0002

Model 1 was not adjusted; Model 2 adjusted for age; and Model 3, based on Model 2, determined direct bilirubin, albumin, smoking status, and drinking status, as well as abdominal obesity indicators (CT-WC) and systemic obesity indicators (BMI), for mutual correction.

**Table 3:** The logistic regression model based on CT measurements of obesity indicators was used to predict the occurrence of MASLD in women.

Variable		Model 1		Model 2		Model 3	
		OR (95% CI)	*p*-value	OR (95% CI)	*p*-value	OR (95% CI)	*p*-value
BMI	Q1	1 (reference)		1 (reference)		1 (reference)	
	Q2	3.19 (2.10, 4.97)	<0.0001	2.90 (1.91, 4.52)	<0.0001	1.63 (1.05, 2.59)	0.0329
	Q3	8.77 (5.97, 13.32)	<0.0001	7.63 (5.18, 11.62)	<0.0001	2.79 (1.81, 4.41)	<0.0001
	Q4	19.05 (13.07, 28.77)	<0.0001	17.01 (11.64, 25.74)	<0.0001	3.14 (1.92, 5.24)	<0.0001
	*P* for trend		<0.0001		<0.0001		<0.0001
	Per SD increment	2.78 (2.50, 3.09)	<0.0001	2.74 (2.47, 3.05)	<0.0001	1.57 (1.31, 1.89)	<0.0001
VFA	Q1	1 (reference)		1 (reference)		1 (reference)	
	Q2	5.53 (2.99, 11.21)	<0.0001	5.76 (3.10, 11.73)	<0.0001	4.24 (2.26, 8.68)	<0.0001
	Q3	24.12 (13.65, 47.47)	<0.0001	25.46 (14.26, 50.49)	<0.0001	14.78 (8.08, 29.81)	<0.0001
	Q4	69.87 (39.76, 136.99)	<0.0001	74.99 (41.95, 148.91)	<0.0001	32.51 (17.31, 66.99)	<0.0001
	*P* for trend		<0.0001		<0.0001		<0.0001
	Per SD increment	3.58 (3.19, 4.03)	<0.0001	3.59 (3.18, 4.06)	<0.0001	2.73 (2.35, 3.18)	<0.0001
SFA	Q1	1 (reference)		1 (reference)		1 (reference)	
	Q2	2.74 (1.94, 3.92)	<0.0001	2.48 (1.75, 3.57)	<0.0001	1.10 (0.75, 1.62)	0.6415
	Q3	4.82 (3.47, 6.81)	<0.0001	4.24 (3.04, 6.01)	<0.0001	1.27 (0.87, 1.89)	0.2203
	Q4	9.47 (6.89, 13.28)	<0.0001	8.31 (6.02, 11.69)	<0.0001	1.22 (0.80, 1.89)	0.3639
	*P* for trend		<0.0001		<0.0001		0.2929
	Per SD increment	2.09 (1.90, 2.29)	<0.0001	2.04 (1.86, 2.24)	<0.0001	0.98 (0.84, 1.14)	0.7782
CT-WC	Q1	1 (reference)		1 (reference)		1 (reference)	
	Q2	4.24 (2.67, 7.01)	<0.0001	3.89 (2.44, 6.46)	<0.0001	2.82 (1.73, 4.77)	<0.0001
	Q3	11.55 (7.49, 18.70)	<0.0001	10.38 (6.70, 16.87)	<0.0001	6.47 (3.97, 10.99)	<0.0001
	Q4	28.45 (18.60, 45.78)	<0.0001	25.24 (16.40, 40.83)	<0.0001	11.51 (6.48, 21.10)	<0.0001
	*P* for trend		<0.0001		<0.0001		<0.0001
	Per SD increment	3.04 (2.73, 3.41)	<0.0001	2.94 (2.63, 3.30)	<0.0001	3.34 (2.60, 4.31)	<0.0001
SMA	Q1	1 (reference)		1 (reference)		1 (reference)	
	Q2	1.45 (1.09, 1.93)	<0.0001	1.52 (1.14, 2.03)	0.0042	1.03 (0.75, 1.41)	0.8570
	Q3	1.71 (1.30, 2.26)	<0.0001	1.90 (1.43, 2.52)	<0.0001	0.96 (0.70, 1.33)	0.8075
	Q4	3.53 (2.73, 4.60)	<0.0001	4.23 (3.24, 5.56)	<0.0001	1.12 (0.80, 1.58)	0.5025
	*P* for trend		<0.0001		<0.0001		0.5750
	Per SD increment	1.76 (1.54, 2.00)	<0.0001	1.94 (1.70, 2.12)	<0.0001	0.96 (0.84, 1.05)	0.3835
L3SMI	Q1	1 (reference)		1 (reference)		1 (reference)	
	Q2	1.31 (0.98, 1.76)	0.0666	1.33 (1.00, 1.79)	0.0536	0.96 (0.70, 1.34)	0.8261
	Q3	1.78 (1.35, 2.36)	<0.0001	1.82 (1.37, 2.41)	<0.0001	1.13 (0.82, 1.56)	0.4622
	Q4	3.91 (3.02, 5.09)	<0.0001	4.22 (3.24, 5.53)	<0.0001	1.25 (0.88, 1.77)	0.2114
	*P* for trend		<0.0001		<0.0001		0.1230
	Per SD increment	1.90 (1.66, 2.08)	<0.0001	1.99 (1.72, 2.11)	<0.0001	0.97 (0.85, 1.06)	0.4662

Model 1 was not adjusted; Model 2 adjusted for age; and Model 3, based on Model 2, determined direct bilirubin, albumin, smoking status, and drinking status, as well as abdominal obesity indicators (CT-WC) and systemic obesity indicators (BMI), for mutual correction.

### Men

In men, all adiposity-related measures were positively associated with MASLD in unadjusted analyses, with clear dose–response trends across quartiles. After full adjustment (Model 3), measures of central adiposity—particularly VFA and CT-WC—remained strong independent correlates of MASLD. Men in the highest VFA quartile had substantially higher odds of MASLD compared with those in the lowest quartile (Q4 vs. Q1: OR = 11.51, 95% CI 8.31–16.13).

CT-WC also showed a robust independent association with MASLD in men, whereas SFA demonstrated a more modest but still statistically significant association. In contrast, generalized obesity as assessed by BMI was not independently associated with MASLD after accounting for fat distribution (per SD OR = 1.06, 95% CI 0.93–1.20). Skeletal muscle indices (SMA and L3SMI) were positively associated with MASLD in univariate analyses but showed substantially attenuated or non-significant associations in fully adjusted models.

Overall, these findings indicate that, among men, central fat accumulation captured by CT-based measures was more strongly associated with MASLD than general obesity or muscle mass alone.

### Women

In women, VFA and CT-WC were also strongly and independently associated with MASLD across all models. Notably, the magnitude of association for VFA was markedly greater in women than in men. Women in the highest VFA quartile had dramatically elevated odds of MASLD compared with those in the lowest quartile (Q4 vs. Q1: OR = 32.51, 95% CI 17.31–66.99).

Unlike in men, BMI remained independently associated with MASLD in women after full adjustment, suggesting that overall adiposity may still contribute to MASLD risk in women even when fat distribution is taken into account. In contrast, SFA did not show an independent association with MASLD in women in the fully adjusted models. Similarly, skeletal muscle indices (SMA and L3SMI) were not significantly associated with MASLD after adjustment.

Taken together, these results highlight pronounced sex-specific patterns: visceral adiposity emerged as the dominant correlate of MASLD in both sexes, but the relative risk gradient associated with increasing VFA was substantially steeper in women. Sensitivity analysis using sex-stratified propensity score matching (1:2 nearest neighbor) showed similar effect estimates for VFA, WC, and other body composition measures, indicating that our main findings were robust to baseline covariate imbalances (Supplementary Table S14). The *E*-value sensitivity analysis further indicated that the strong associations observed for VFA and CT-WC were relatively robust to potential unmeasured confounding (e.g., the *E*-value for the highest percentile of VFA in females was 64.52; see Supplementary Tables S15, S16).

### Nonlinear dose–response relationships and potential threshold effects

**Figure 3:**
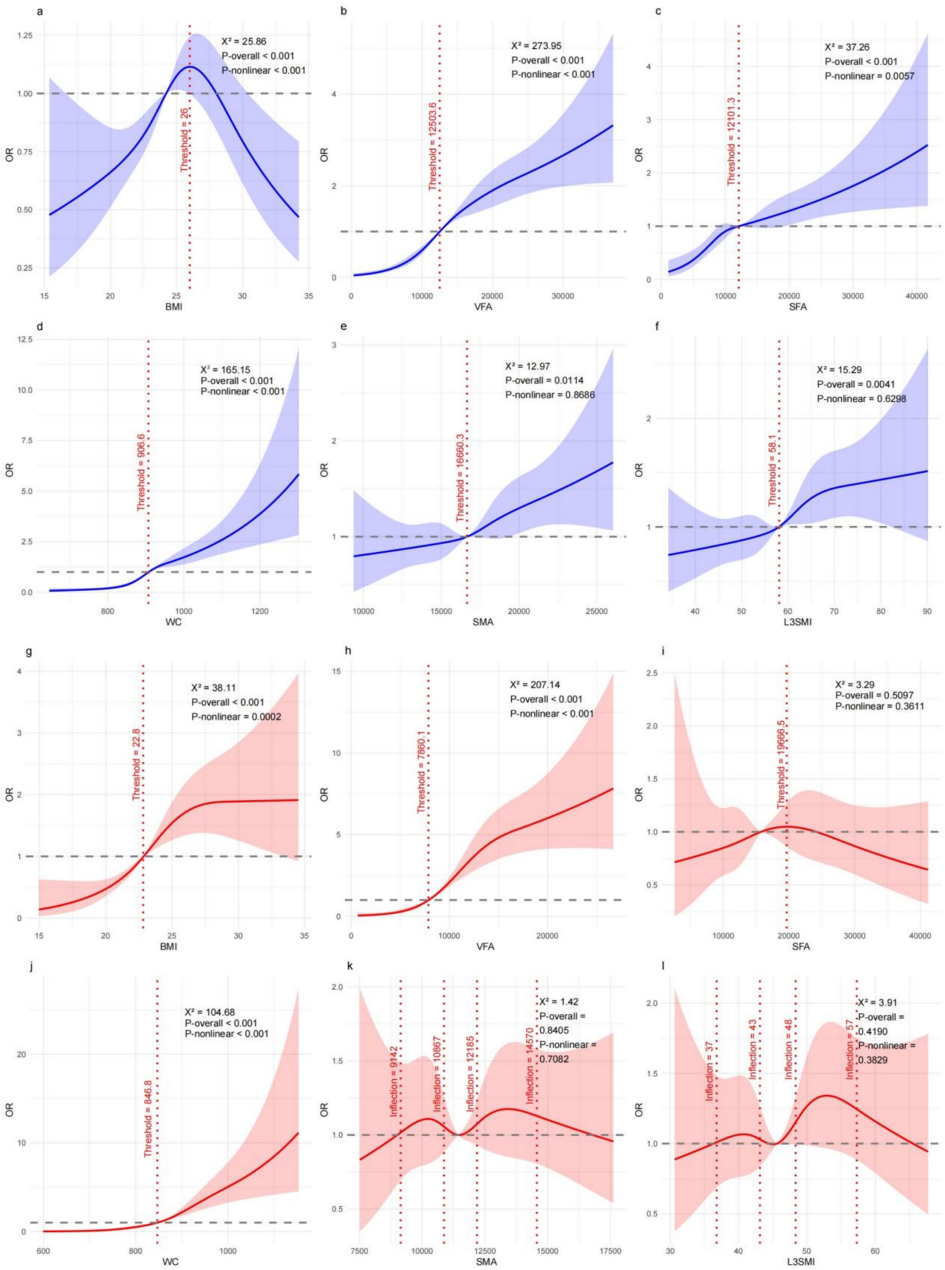
Nonlinear associations between CT-derived obesity indices and MASLD using restricted cubic spline models, stratified by sex. **(a–f)** Males: **(a)** BMI, **(b)** VFA, **(c)** SFA, **(d)** WC, **(e)** SMA, and **(f)** L3SMI. **(g–l)** Females: **(g)** BMI, **(h)** VFA, **(i)** SFA, **(j)** WC, **(k)** SMA, and **(l)** L3SMI.

To further characterize the shape of these associations, we examined dose–response relationships using restricted cubic spline models (Figure 3).

In men, VFA exhibited a clear non-linear association with MASLD odds. At lower levels of visceral fat, MASLD risk increased slowly; beyond an apparent threshold (approximately 12503.6 mm^2^), the odds of MASLD rose sharply, followed by a tendency toward plateauing at very high VFA levels. A similar non-linear pattern was observed for CT-WC, whereas SFA showed a weaker and less pronounced curvature. In contrast, skeletal muscle indices demonstrated largely linear or flat relationships with MASLD risk.

In women, non-linear associations were also evident for VFA and CT-WC. MASLD odds increased gradually at moderate levels of visceral fat but escalated steeply at higher levels, with an apparent inflection point around 7860.1 mm^2^ for VFA. SFA and skeletal muscle measures did not show meaningful non-linear associations with MASLD in women.

These spline analyses indicate that MASLD risk does not increase uniformly across the range of adiposity but instead accelerates once visceral fat exceeds certain levels, underscoring the potential clinical relevance of identifying high-risk zones rather than relying on linear assumptions.

### Discrimination performance and subgroup analyses

We next evaluated whether incorporating CT-derived body composition measures improved discrimination of MASLD compared with BMI alone. Receiver operating characteristic (ROC) analyses demonstrated that models including CT-derived central adiposity measures (with or without muscle indices) achieved higher discrimination than BMI-only models in both sexes.

In men, the area under the ROC curve (AUC) increased from [AUC = 0.707 (95% CI: 0.545–0.769)] for the BMI-only model to [AUC = 0.758 (95% CI: 0.580–0.821)] when CT-derived fat (and muscle) measures were included. In women, discrimination similarly improved from [AUC = 0.774 (95% CI: 0.631–0.789)] to [AUC = 0.833 (95% CI: 0.746–0.783)] with the addition of CT-based measures. These findings suggest that accounting for fat distribution provides incremental value for MASLD risk discrimination beyond conventional anthropometric indices. Further details are shown in Supplementary Figures S1, S2.

Subgroup analyses stratified by age (<50 vs. ≥50 years) and metabolic comorbidities (hypertension, dyslipidemia, type 2 diabetes, and hyperuricemia) showed consistent associations between VFA and MASLD, with no strong evidence of effect modification. Sensitivity analyses yielded effect estimates comparable to the primary analyses, supporting the robustness of the observed associations (Details are provided in Supplementary Tables S2–S5). Similar results were observed after restricting the CT-laboratory interval to ≤30 and ≤90 days, indicating that the findings were not materially affected by measurement timing (see Supplementary Tables S18, S19).

## Discussion

### Summary of key findings

In this large cross-sectional study of Chinese adults, we systematically examined the associations between CT-derived body composition measures and MASLD using sex-stratified, non-linear analytical approaches. Several key findings emerged. First, visceral adiposity quantified by CT was the strongest correlate of MASLD in both men and women, consistently outperforming BMI after adjustment for fat distribution and metabolic covariates. Second, the strength and shape of this association differed markedly by sex, with women exhibiting a substantially steeper risk gradient at higher levels of visceral fat. Third, the relationships between central adiposity and MASLD were distinctly non-linear, suggesting potential threshold effects rather than a constant linear increase in risk. In contrast, skeletal muscle quantity alone was not independently associated with MASLD once adiposity was accounted for. Together, these findings underscore the dominant role of fat distribution—particularly visceral fat—in MASLD risk stratification and highlight important sex-specific and non-linear patterns that are not captured by conventional anthropometric indices.

### Comparison with previous studies

The close relationship between obesity and MASLD has been well established (17). Our findings extend prior evidence showing that visceral adiposity is more strongly associated with fatty liver disease than general obesity defined by BMI (18, 19). Unlike BMI, which does not distinguish between lean and fat mass or reflect regional fat distribution, visceral fat represents a metabolically active depot that directly drains into the portal circulation (20), thereby promoting hepatic insulin resistance, triglyceride accumulation, and lipotoxic injury (21, 22). This provides biological plausibility for the stronger association observed between visceral fat and MASLD (23).

By leveraging CT-based measurements, our study provides objective quantification of visceral and subcutaneous fat compartments and confirms that central adiposity conveys risk information beyond BMI. Importantly, this association persisted after mutual adjustment for BMI and other adiposity measures, indicating that visceral fat is not merely a surrogate for general obesity (24, 25). These findings are particularly relevant in Asian populations, where individuals may develop substantial visceral fat and metabolic risk at relatively low BMI levels. Within the context of the updated MASLD definition, our results reinforce the limitations of BMI-based risk assessment and support a more nuanced evaluation of adiposity distribution.

Regarding sex differences, our results align with previous epidemiological observations that women experience a disproportionate increase in metabolic and hepatic risk once visceral fat accumulation exceeds certain levels (26, 27). Furthermore, the modest association between subcutaneous fat and MASLD in men—but not in women—is consistent with studies suggesting that subcutaneous adipose tissue may serve as a relatively protective energy buffer, particularly in women (27–30).

In contrast to adiposity measures, skeletal muscle quantity assessed by CT (SMA and L3SMI) was not independently associated with MASLD after accounting for fat distribution (31, 32). This finding may appear discordant with prior studies reporting links between sarcopenia and NAFLD (33, 34). Several factors may explain this discrepancy. First, many previous studies relied on indirect measures of muscle mass or did not adequately adjust for visceral adiposity, which may confound observed associations. Second, our analysis focused on muscle quantity rather than muscle quality or function; intramuscular fat infiltration (myosteatosis) and muscle strength may be more relevant to metabolic risk than muscle area alone (35). Third, in populations with substantial adiposity, the adverse metabolic effects of visceral fat may dominate and mask any protective influence of muscle mass (36).

These considerations suggest that muscle-related risk in MASLD may be context-dependent and more evident in specific phenotypes, such as sarcopenic obesity, rather than through low muscle mass alone. Future studies incorporating muscle density, strength, and longitudinal changes may help clarify the role of skeletal muscle in MASLD pathogenesis.

### Clinical and research implications

From a clinical perspective, our findings emphasize that assessment of fat distribution provides important information beyond BMI for identifying individuals at risk of MASLD. Reliance on BMI alone may miss individuals with relatively normal body weight but disproportionately high visceral fat, as well as overestimate risk in those with predominantly subcutaneous fat accumulation.

At the population level, the observed non-linear and sex-specific patterns highlight the potential value of targeted prevention strategies that focus on limiting visceral fat accumulation rather than weight reduction alone. However, given the cross-sectional nature of the study, these implications should be interpreted as hypothesis-generating rather than prescriptive.

### Strengths and limitations

This study has several strengths, including a large sample size, objective CT-based quantification of body composition, application of the updated MASLD definition, and use of sex-stratified and non-linear analytical approaches. Nevertheless, several limitations warrant consideration. First, the cross-sectional design precludes causal inference and does not allow assessment of temporal relationships. Second, MASLD was defined based on imaging rather than liver biopsy, which may have resulted in under-detection of mild steatosis. Third, as this was a hospital-based cross-sectional study, generalizability to the broader community may be limited. Although propensity score matching was used as a sensitivity analysis to balance measured covariates, residual unmeasured confounding cannot be fully excluded. Fourth, laboratory measurements—including ALT, AST, and triglycerides—were extracted from results closest to the CT date within a 6-month window, which may introduce temporal discordance. In addition, data on lipid-lowering medication use were incomplete, preventing precise adjustment for treatment effects. These limitations may have influenced MASLD classification and are acknowledged accordingly. Given the retrospective cross-sectional design and the 6-month alignment window, some temporal discordance between CT and laboratory measurements is possible. Lipid-lowering therapy initiated during this interval could reduce triglyceride levels below the diagnostic threshold, potentially leading to false-negative MASLD classification. Such misclassification would be expected to attenuate associations toward the null, suggesting that our estimates are more likely conservative than inflated.

Future research should prioritize longitudinal and interventional studies to clarify causal pathways linking visceral fat accumulation to liver injury and to determine whether targeted reduction of visceral adiposity leads to meaningful improvements in MASLD outcomes. In addition, future studies incorporating CT-derived measures of muscle quality, such as muscle radiodensity, may provide complementary information beyond muscle quantity alone. Given the pronounced sex differences observed, studies incorporating hormonal status and reproductive factors may further refine risk stratification and inform sex-specific prevention strategies.

## Conclusion

In conclusion, this cross-sectional study indicates that CT-derived measures of abdominal fat distribution are strongly associated with MASLD and may provide incremental value beyond conventional BMI-based assessment. Visceral adiposity, quantified by CT-based VFA and WC, was more closely associated with MASLD risk than generalized obesity measured by BMI in both men and women. Notably, we observed sex-specific and non-linear association patterns, with a steeper increase in MASLD risk at higher visceral fat levels among women and a comparatively stronger association with subcutaneous fat among men.

These findings highlight the potential importance of fat distribution, rather than total adiposity alone, in MASLD risk stratification. From a clinical perspective, our results suggest that incorporating central obesity indicators, such as WC or imaging-derived fat measures, could complement BMI in identifying individuals at higher MASLD risk. However, given the cross-sectional design, causal inferences cannot be made, and longitudinal and interventional studies are needed to confirm these associations and clarify their clinical implications.

## Data Availability

The raw data supporting the conclusions of this article will be made available by the authors, without undue reservation.
