# Supplementary material for: CT-assessed abdominal visceral adiposity and MASLD: a sex-stratified cross-sectional analysis
Source: Front Nutr. 2026 Mar 25;13:1750470. doi: 10.3389/fnut.2026.1750470 (PMC13056823; doi:10.3389/fnut.2026.1750470)
Supplement: Supplementary file 1 [file Table_1.docx]

**Supplementary Materials**

[Supplemental Table 1. Sensitivity Analyses of Quantitative and Categorical Metabolic Abnormalities in Relation to MASLD in Men 2](#_Toc27386)

[Supplemental Table 3. Logistic Regression of Body Composition Indices and MASLD in Age Subgroups among Men(<50 vs ≥50 Years) 4](#_Toc18011)

[Supplemental Table 4. Logistic Regression of Body Composition Indices and MASLD in Age Subgroups among Women(<50 vs ≥50 Years) 5](#_Toc1928)

[Supplemental Table 5. Logistic Regression of Body Composition Indices and MASLD in Hypertension Subgroups Among Men(With vs Without Hypertension) 6](#_Toc11910)

[Supplemental Table 6. Logistic Regression of Body Composition Indices and MASLD in Hypertension Subgroups Among Women(With vs Without Hypertension) 7](#_Toc3246)

[Supplemental Table 7. Logistic Regression of Body Composition Indices and MASLD in Hypertension Subgroups Among Men(With vs Without Hyperlipidemia) 8](#_Toc192)

[Supplemental Table 8. Logistic Regression of Body Composition Indices and MASLD in Hypertension Subgroups Among Women(With vs Without Hyperlipidemia) 9](#_Toc8469)

[Supplemental Table 9. Logistic Regression of Body Composition Indices and MASLD in Hypertension Subgroups Among Men(With vs Without Diabetes) 10](#_Toc4581)

[Supplemental Table 10. Logistic Regression of Body Composition Indices and MASLD in Hypertension Subgroups Among Women(With vs Without Diabetes) 11](#_Toc13241)

[Supplemental Table 11. Logistic Regression of Body Composition Indices and MASLD in Hypertension Subgroups Among Men(With vs Without Hyperuricemia) 12](#_Toc29793)

[Supplemental Table 12. Logistic Regression of Body Composition Indices and MASLD in Hypertension Subgroups Among Women(With vs Without Hyperuricemia) 13](#_Toc30511)

[Supplemental Table 13. Sex-Stratified Propensity Score Matching Results (Model 3) 14](#_Toc15081)

[Supplemental Figure 1. ROC Curves Comparing Predictive Performance of VFA, SFA, BMI, WC,SMA and L3SMI for MASLD in Men. 15](#_Toc31741)

[Supplemental Figure 2. ROC Curves Comparing Predictive Performance of VFA, SFA, BMI, WC,SMA and L3SMI for MASLD in Women. 16](#_Toc489)

**Supplemental Table 1. Sensitivity Analyses of Quantitative and Categorical Metabolic Abnormalities in Relation to MASLD in Men**

| Variable | | Model4 | | Model5 | |
| --- | --- | --- | --- | --- | --- |
|  |  | OR (95% CI) | P-value | OR (95% CI) | P-value |
| BMI | Q1 | 1(reference) |  | 1(reference) |  |
|  | Q2 | 1.67(1.28,2.19) | 0.0002 | 1.70(1.31,2.23) | <0.0001 |
|  | Q3 | 2.12(1.61,2.81) | <0.0001 | 2.19(1.67,2.89) | <0.0001 |
|  | Q4 | 1.66(1.20,2.29) | 0.0020 | 1.75(1.27,2.41) | 0.0006 |
| VFA | Q1 | 1(reference) |  | 1(reference) |  |
|  | Q2 | 2.74(2.02,3.75) | <0.0001 | 3.15(2.33,4.31) | <0.0001 |
|  | Q3 | 4.57(3.34,6.34) | <0.0001 | 5.73(4.21,7.90) | <0.0001 |
|  | Q4 | 5.86(4.14,8.38) | <0.0001 | 7.92(5.66,11.21) | <0.0001 |
| SFA | Q1 | 1(reference) |  | 1(reference) |  |
|  | Q2 | 1.58(1.23,2.04) | 0.0004 | 1.59(1.24,2.06) | 0.0003 |
|  | Q3 | 1.54(1.19,2.02) | 0.0012 | 1.54(1.18,2.00) | 0.0013 |
|  | Q4 | 2.13(1.59,2.85) | <0.0001 | 2.02(1.52,2.70) | <0.0001 |
| WC | Q1 | 1(reference) |  | 1(reference) |  |
|  | Q2 | 1.78(1.34,2.39) | <0.0001 | 2.08(1.57,2.78) | <0.0001 |
|  | Q3 | 2.90(2.13,3.97) | <0.0001 | 3.60(2.65,4.91) | <0.0001 |
|  | Q4 | 3.03(2.07,4.44) | <0.0001 | 4.07(2.80,5.93) | <0.0001 |
| SMA | Q1 | 1(reference) |  | 1(reference) |  |
|  | Q2 | 1.08(0.87,1.35) | 0.4774 | 1.10(0.89,1.38) | 0.3745 |
|  | Q3 | 1.10(0.88,1.38) | 0.3935 | 1.12(0.90,1.41) | 0.2919 |
|  | Q4 | 1.32(1.03,1.69) | 0.0257 | 1.38(1.08,1.76) | 0.0096 |
| L3SMI | Q1 | 1(reference) |  | 1(reference) |  |
|  | Q2 | 1.07(0.86,1.34) | 0.5373 | 1.10(0.88,1.37) | 0.4144 |
|  | Q3 | 1.20(0.96,1.51) | 0.1057 | 1.24(0.99,1.55) | 0.0634 |
|  | Q4 | 1.36(1.06,1.74) | 0.0152 | 1.43(1.12,1.84) | 0.0040 |

**Model 4 adds quantitative indicators of hypertension, diabetes, hyperlipidemia, and hyperuricemia to Model 3. Model 5 adds qualitative indicators of hypertension, diabetes, hyperlipidemia, and hyperuricemia to Model 3.**

**Supplemental Table 2. Sensitivity Analyses of Quantitative and Categorical Metabolic Abnormalities in Relation to MASLD in Women**

| Variable | | Model4 | | Model5 | |
| --- | --- | --- | --- | --- | --- |
|  |  | OR (95% CI) | P-value | OR (95% CI) | P-value |
| BMI | Q1 | 1(reference) |  | 1(reference) |  |
|  | Q2 | 1.57(1.00,2.52) | 0.0527 | 1.60(1.02,2.55) | 0.0442 |
|  | Q3 | 2.51(1.61,4.00) | <0.0001 | 2.70(1.74,4.29) | <0.0001 |
|  | Q4 | 2.53(1.52,4.28) | 0.0004 | 2.79(1.69,4.71) | <0.0001 |
| VFA | Q1 | 1(reference) |  | 1(reference) |  |
|  | Q2 | 3.39(1.79,6.98) | 0.0004 | 3.85(2.04,7.91) | <0.0001 |
|  | Q3 | 9.86(5.32,20.08) | <0.0001 | 12.37(6.73,25.04) | <0.0001 |
|  | Q4 | 17.59(9.16,36.82) | <0.0001 | 24.13(12.74,50.02) | <0.0001 |
| SFA | Q1 | 1(reference) |  | 1(reference) |  |
|  | Q2 | 1.13(0.76,1.70) | 0.5443 | 1.13(0.76,1.69) | 0.5391 |
|  | Q3 | 1.43(0.96,2.15) | 0.0816 | 1.37(0.93,2.05) | 0.1179 |
|  | Q4 | 1.40(0.90,2.21) | 0.1432 | 1.33(0.85,2.08) | 0.2143 |
| WC | Q1 | 1(reference) |  | 1(reference) |  |
|  | Q2 | 2.36(1.43,4.04) | 0.0012 | 2.56(1.56,4.35) | 0.0003 |
|  | Q3 | 4.78(2.88,8.23) | <0.0001 | 5.70(3.47,9.75) | <0.0001 |
|  | Q4 | 6.68(3.66,12.54) | <0.0001 | 8.49(4.71,15.76) | <0.0001 |
| SMA | Q1 | 1(reference) |  | 1(reference) |  |
|  | Q2 | 1.05(0.76,1.45) | 0.7814 | 1.07(0.78,1.48) | 0.6795 |
|  | Q3 | 0.95(0.68,1.33) | 0.7637 | 0.97(0.69,1.35) | 0.8405 |
|  | Q4 | 1.13(0.80,1,62) | 0.4837 | 1.11(0.79,1.58) | 0.5505 |
| L3SMI | Q1 | 1(reference) |  | 1(reference) |  |
|  | Q2 | 0.95(0.68,1.33) | 0.7720 | 0.99(0.71,1.38) | 0.9582 |
|  | Q3 | 1.11(0.80,1.56) | 0.5289 | 1.12(0.81,1.56) | 0.4951 |
|  | Q4 | 1.28(0.89,1.83) | 0.1817 | 1.28(0.89,1.82) | 0.1806 |

**Model 4 adds quantitative indicators of hypertension, diabetes, hyperlipidemia, and hyperuricemia to Model 3. Model 5 adds qualitative indicators of hypertension, diabetes, hyperlipidemia, and hyperuricemia to Model 3.**

**Supplemental Table 3. Logistic Regression of Body Composition Indices and MASLD in Age Subgroups among Men(<50 vs ≥50 Years)**

| Group | Exposure |  | MASLD | NO-MASLD | OR(95% CI) | P-value | p for interaction |
| --- | --- | --- | --- | --- | --- | --- | --- |
| <50 | BMI | Q1 | 52 | 557 |  |  | 0.8182 |
|  |  | Q2 | 149 | 467 | 1.72(1.19,2.51) | 0.0047 |  |
|  |  | Q3 | 229 | 368 | 2.16(1.48,3.20) | <0.0001 |  |
|  |  | Q4 | 310 | 297 | 1.69(1.08,2.64) | 0.0229 |  |
|  | VFA | Q1 | 34 | 574 |  |  | 0.1976 |
|  |  | Q2 | 124 | 483 | 2.84(1.89,4.37) | <0.0001 |  |
|  |  | Q3 | 254 | 353 | 6.73(4.47,10.39) | <0.0001 |  |
|  |  | Q4 | 328 | 279 | 8.70(5.59,13.84) | <0.0001 |  |
|  | SFA | Q1 | 55 | 553 |  |  | 0.7355 |
|  |  | Q2 | 149 | 458 | 1.54(1.07,2.23) | 0.0202 |  |
|  |  | Q3 | 219 | 388 | 1.90(1.31,2.80) | 0.0009 |  |
|  |  | Q4 | 317 | 290 | 2.51(1.66,3.84) | <0.0001 |  |
|  | WC | Q1 | 38 | 570 |  |  | 0.1200 |
|  |  | Q2 | 118 | 489 | 2.55(1.70,3.90) | <0.0001 |  |
|  |  | Q3 | 248 | 359 | 6.04(3.92,9.47) | <0.0001 |  |
|  |  | Q4 | 336 | 271 | 7.33(4.28,12.73) | <0.0001 |  |
|  | SMA | Q1 | 101 | 507 |  |  | 0.3976 |
|  |  | Q2 | 149 | 458 | 1.22(0.90,1.66) | 0.2067 |  |
|  |  | Q3 | 202 | 405 | 1.30(0.95,1.78) | 0.1055 |  |
|  |  | Q4 | 288 | 319 | 1.57(1.12,2.19) | 0.0085 |  |
|  | L3SMI | Q1 | 102 | 506 |  |  | 0.0360 |
|  |  | Q2 | 154 | 453 | 1.13(0.83,1.54) | 0.4508 |  |
|  |  | Q3 | 195 | 412 | 1.23(0.90,1.69) | 0.2008 |  |
|  |  | Q4 | 289 | 318 | 1.69(1.20,2.38) | 0.0029 |  |
| ≥50 | BMI | Q1 | 50 | 559 |  |  |  |
|  |  | Q2 | 131 | 475 | 1.89(1.31,2.76) | 0.0008 |  |
|  |  | Q3 | 197 | 410 | 2.28(1.55,3.39) | <0.0001 |  |
|  |  | Q4 | 251 | 356 | 2.09(1.33,3.31) | 0.0016 |  |
|  | VFA | Q1 | 37 | 571 |  |  |  |
|  |  | Q2 | 109 | 498 | 3.00(2.01,4.57) | <0.0001 |  |
|  |  | Q3 | 199 | 408 | 6.10(4.08,9.32) | <0.0001 |  |
|  |  | Q4 | 284 | 323 | 9.91(6.43,15.60) | <0.0001 |  |
|  | SFA | Q1 | 67 | 541 |  |  |  |
|  |  | Q2 | 150 | 457 | 1.39(1.00,1.96) | 0.0540 |  |
|  |  | Q3 | 178 | 429 | 1.39(0.99,1.98) | 0.0617 |  |
|  |  | Q4 | 234 | 373 | 1.52(1.04,2.22) | 0.0301 |  |
|  | WC | Q1 | 46 | 562 |  |  |  |
|  |  | Q2 | 124 | 483 | 2.54(1.74,3.76) | <0.0001 |  |
|  |  | Q3 | 202 | 405 | 4.14(2.76,6.29) | <0.0001 |  |
|  |  | Q4 | 257 | 350 | 4.83(3.00,7.89) | <0.0001 |  |
|  | SMA | Q1 | 93 | 515 |  |  |  |
|  |  | Q2 | 154 | 453 | 1.32(0.98,1.81) | 0.0732 |  |
|  |  | Q3 | 154 | 453 | 1.12(0.82,1.55) | 0.4732 |  |
|  |  | Q4 | 228 | 379 | 1.54(1.10,2.16) | 0.0113 |  |
|  | L3SMI | Q1 | 103 | 505 |  |  |  |
|  |  | Q2 | 147 | 460 | 1.30(0.96,1.76) | 0.0960 |  |
|  |  | Q3 | 163 | 444 | 1.25(0.92,1.72) | 0.1532 |  |
|  |  | Q4 | 216 | 391 | 1.43(1.02,2.01) | 0.0388 |  |

**Supplemental Table 4. Logistic Regression of Body Composition Indices and MASLD in Age Subgroups among Women(<50 vs ≥50 Years)**

| Group | Exposure |  | MASLD | NO-MASLD | OR(95% CI) | P-value | p for interaction |
| --- | --- | --- | --- | --- | --- | --- | --- |
| <50 | BMI | Q1 | 13 | 342 |  |  | 0.1034 |
|  |  | Q2 | 16 | 341 | 0.67(0.31,1.48) | 0.3127 |  |
|  |  | Q3 | 58 | 295 | 1.59(0.81,3.30) | 0.1918 |  |
|  |  | Q4 | 135 | 219 | 1.91(0.87,4.38) | 0.1147 |  |
|  | VFA | Q1 | 3 | 352 |  |  | 0.5537 |
|  |  | Q2 | 14 | 341 | 3.59(1.15,15.76) | 0.0476 |  |
|  |  | Q3 | 56 | 298 | 12.50(4.42,52.44) | <0.0001 |  |
|  |  | Q4 | 149 | 206 | 33.33(11.54,141.54) | <0.0001 |  |
|  | SFA | Q1 | 14 | 341 |  |  | 0.6169 |
|  |  | Q2 | 34 | 321 | 1.04(0.53,2.12) | 0.9108 |  |
|  |  | Q3 | 48 | 306 | 0.87(0.44,1.79) | 0.6907 |  |
|  |  | Q4 | 126 | 229 | 1.30(0.61,2.86) | 0.5053 |  |
|  | WC | Q1 | 7 | 348 |  |  | 0.4966 |
|  |  | Q2 | 25 | 330 | 2.74(1.21,7.05) | 0.0226 |  |
|  |  | Q3 | 46 | 308 | 4.21(1.88,10.75) | 0.0011 |  |
|  |  | Q4 | 144 | 211 | 12.30(5.03,33.64) | <0.0001 |  |
|  | SMA | Q1 | 30 | 325 |  |  | 0.7595 |
|  |  | Q2 | 37 | 318 | 0.81(0.47,1.41) | 0.4538 |  |
|  |  | Q3 | 51 | 304 | 0.84(0.48,1.46) | 0.5237 |  |
|  |  | Q4 | 104 | 250 | 0.91(0.51,1.63) | 0.7490 |  |
|  | L3SMI | Q1 | 31 | 324 |  |  | 0.0523 |
|  |  | Q2 | 31 | 324 | 0.74(0.41,1.31) | 0.2957 |  |
|  |  | Q3 | 45 | 309 | 0.82(0.47,1.44) | 0.4921 |  |
|  |  | Q4 | 115 | 240 | 1.18(0.66,2.14) | 0.5761 |  |
| ≥50 | BMI | Q1 | 19 | 364 |  |  |  |
|  |  | Q2 | 85 | 296 | 3.38(2.00,5.96) | <0.0001 |  |
|  |  | Q3 | 126 | 257 | 4.33(2.52,7.75) | <0.0001 |  |
|  |  | Q4 | 192 | 189 | 5.99(3.24,11.50) | <0.0001 |  |
|  | VFA | Q1 | 13 | 369 |  |  |  |
|  |  | Q2 | 61 | 321 | 4.37(2.41,8.51) | <0.0001 |  |
|  |  | Q3 | 138 | 244 | 11.05(6.19,21.32) | <0.0001 |  |
|  |  | Q4 | 210 | 172 | 17.96(9.81,35.39) | <0.0001 |  |
|  | SFA | Q1 | 45 | 337 |  |  |  |
|  |  | Q2 | 95 | 287 | 1.22(0.79,1.87) | 0.3721 |  |
|  |  | Q3 | 122 | 260 | 1.25(0.81,1.93) | 0.3202 |  |
|  |  | Q4 | 160 | 222 | 1.00(0.62,1.64) | 0.9966 |  |
|  | WC | Q1 | 24 | 358 |  |  |  |
|  |  | Q2 | 74 | 308 | 3.07(1.86,5.23) | <0.0001 |  |
|  |  | Q3 | 135 | 247 | 6.27(3.72,10.87) | <0.0001 |  |
|  |  | Q4 | 189 | 193 | 9.29(4.89,18.05) | <0.0001 |  |
|  | SMA | Q1 | 60 | 322 |  |  |  |
|  |  | Q2 | 98 | 284 | 1.30(0.88,1.94) | 0.1923 |  |
|  |  | Q3 | 110 | 272 | 1.09(0.73,1.64) | 0.6711 |  |
|  |  | Q4 | 154 | 228 | 1.14(0.75,1.75) | 0.5369 |  |
|  | L3SMI | Q1 | 61 | 321 |  |  |  |
|  |  | Q2 | 86 | 296 | 1.06(0.71,1.59) | 0.7815 |  |
|  |  | Q3 | 116 | 266 | 1.43(0.96,2.13) | 0.0785 |  |
|  |  | Q4 | 159 | 223 | 1.28(0.83,1.98) | 0.2629 |  |

**Supplemental Table 5. Logistic Regression of Body Composition Indices and MASLD in Hypertension Subgroups Among Men(With vs Without Hypertension)**

| Group | Exposure |  | MASLD | NO-MASLD | OR(95% CI) | P-value | p for interaction |
| --- | --- | --- | --- | --- | --- | --- | --- |
| Hypertension | BMI | Q1 | 77 | 541 |  |  | 0.8182 |
|  |  | Q2 | 194 | 423 | 2.05(1.49,2.83) | <0.0001 |  |
|  |  | Q3 | 241 | 379 | 2.06(1.47,2.87) | <0.0001 |  |
|  |  | Q4 | 316 | 298 | 1.89(1.29,2.78) | 0.0012 |  |
|  | VFA | Q1 | 45 | 573 |  |  | 0.1976 |
|  |  | Q2 | 185 | 432 | 4.30(2.98,6.21) | <0.0001 |  |
|  |  | Q3 | 261 | 356 | 6.86(4.72,9.97) | <0.0001 |  |
|  |  | Q4 | 337 | 280 | 9.56(6.40,14.28) | <0.0001 |  |
|  | SFA | Q1 | 89 | 530 |  |  | 0.7355 |
|  |  | Q2 | 189 | 427 | 1.54(1.14,2.09) | 0.0053 |  |
|  |  | Q3 | 235 | 382 | 1.60(1.17,2.20) | 0.0036 |  |
|  |  | Q4 | 315 | 302 | 1.85(1.30,2.61) | 0.0005 |  |
|  | WC | Q1 | 58 | 560 |  |  | 0.1200 |
|  |  | Q2 | 179 | 438 | 3.20(2.27,4.52) | <0.0001 |  |
|  |  | Q3 | 245 | 372 | 4.46(3.07,6.48) | <0.0001 |  |
|  |  | Q4 | 346 | 271 | 6.51(4.13,10.25) | <0.0001 |  |
|  | SMA | Q1 | 135 | 483 |  |  | 0.3976 |
|  |  | Q2 | 173 | 444 | 1.05(0.79,1.39) | 0.7400 |  |
|  |  | Q3 | 216 | 401 | 1.09(0.82,1.46) | 0.5362 |  |
|  |  | Q4 | 304 | 313 | 1.27(0.93,1.75) | 0.1323 |  |
|  | L3SMI | Q1 | 132 | 486 |  |  | 0.0360 |
|  |  | Q2 | 186 | 431 | 1.26(0.95,1.67) | 0.1084 |  |
|  |  | Q3 | 224 | 393 | 1.30(0.98,1.74) | 0.0726 |  |
|  |  | Q4 | 286 | 331 | 1.28(0.93,1.76) | 0.1247 |  |
| No-Hypertension | BMI | Q1 | 43 | 556 |  |  |  |
|  |  | Q2 | 90 | 506 | 1.22(0.81,1.85) | 0.3377 |  |
|  |  | Q3 | 167 | 438 | 1.59(1.03,2.44) | 0.0350 |  |
|  |  | Q4 | 241 | 348 | 1.48(0.89,2.47) | 0.1307 |  |
|  | VFA | Q1 | 21 | 577 |  |  |  |
|  |  | Q2 | 82 | 515 | 3.60(2.14,6.05) | <0.0001 |  |
|  |  | Q3 | 188 | 409 | 9.93(5.89,16.77) | <0.0001 |  |
|  |  | Q4 | 250 | 347 | 13.95(7.95,24.50) | <0.0001 |  |
|  | SFA | Q1 | 36 | 562 |  |  |  |
|  |  | Q2 | 106 | 491 | 1.74(1.13,2.67) | 0.0116 |  |
|  |  | Q3 | 159 | 438 | 2.08(1.34,3.24) | 0.00115 |  |
|  |  | Q4 | 240 | 357 | 2.80(1.72,4.57) | <0.0001 |  |
|  | WC | Q1 | 33 | 565 |  |  |  |
|  |  | Q2 | 68 | 529 | 1.83(1.15,2.92) | 0.0109 |  |
|  |  | Q3 | 179 | 418 | 5.36(3.33,8.64) | <0.0001 |  |
|  |  | Q4 | 261 | 336 | 7.95(4.49,14.09) | <0.0001 |  |
|  | SMA | Q1 | 80 | 518 |  |  |  |
|  |  | Q2 | 107 | 490 | 1.00(0.71,1.41) | 0.9819 |  |
|  |  | Q3 | 134 | 463 | 1.06(0.74,1.50) | 0.7592 |  |
|  |  | Q4 | 220 | 377 | 1.72(1.19,2.50) | 0.0041 |  |
|  | L3SMI | Q1 | 82 | 516 |  |  |  |
|  |  | Q2 | 110 | 487 | 1.09(0.78,1.53) | 0.6211 |  |
|  |  | Q3 | 130 | 467 | 1.06(0.74,1.50) | 0.7556 |  |
|  |  | Q4 | 219 | 378 | 2.00(1.38,2.91) | 0.0003 |  |

**Supplemental Table 6. Logistic Regression of Body Composition Indices and MASLD in Hypertension Subgroups Among Women(With vs Without Hypertension)**

| Group | Exposure |  | MASLD | NO-MASLD | OR(95% CI) | P-value | p for interaction |
| --- | --- | --- | --- | --- | --- | --- | --- |
| Hypertension | BMI | Q1 | 23 | 260 |  |  | 0.2755 |
|  |  | Q2 | 77 | 206 | 3.04(1.78,5.21) | <0.0001 |  |
|  |  | Q3 | 102 | 175 | 3.44(1.94,6.11) | <0.0001 |  |
|  |  | Q4 | 158 | 122 | 6.08(3.05,12.09) | <0.0001 |  |
|  | VFA | Q1 | 18 | 263 |  |  | 0.3586 |
|  |  | Q2 | 55 | 226 | 2.88(1.61,5.13) | 0.0003 |  |
|  |  | Q3 | 121 | 159 | 7.43(4.19,13.20) | <0.0001 |  |
|  |  | Q4 | 166 | 115 | 11.37(6.13,21.09) | <0.0001 |  |
|  | SFA | Q1 | 47 | 234 |  |  | 0.3759 |
|  |  | Q2 | 74 | 207 | 0.97(0.62,1.52) | 0.8924 |  |
|  |  | Q3 | 110 | 170 | 1.16(0.73,1.84) | 0.5208 |  |
|  |  | Q4 | 129 | 152 | 0.86(0.51,1.47) | 0.5923 |  |
|  | WC | Q1 | 26 | 255 |  |  | 0.4557 |
|  |  | Q2 | 69 | 212 | 2.76(1.64,4.65) | 0.0001 |  |
|  |  | Q3 | 108 | 172 | 4.82(2.74,8.48) | <0.0001 |  |
|  |  | Q4 | 157 | 124 | 8.25(4.04,16.85) | <0.0001 |  |
|  | SMA | Q1 | 60 | 221 |  |  | 0.0645 |
|  |  | Q2 | 71 | 210 | 0.97(0.63,1.48) | 0.8723 |  |
|  |  | Q3 | 101 | 179 | 1.28(0.83,1.96) | 0.2609 |  |
|  |  | Q4 | 128 | 153 | 0.92(0.57,1.49) | 0.7247 |  |
|  | L3SMI | Q1 | 64 | 217 |  |  | 0.4463 |
|  |  | Q2 | 71 | 210 | 1.02(0.66,1.56) | 0.9300 |  |
|  |  | Q3 | 98 | 182 | 1.14(0.74,1.75) | 0.5547 |  |
|  |  | Q4 | 127 | 154 | 0.92(0.57,1.51) | 0.7479 |  |
| No-Hypertension | BMI | Q1 | 11 | 448 |  |  |  |
|  |  | Q2 | 33 | 420 | 1.43(0.69,2.96) | 0.3402 |  |
|  |  | Q3 | 81 | 378 | 2.41(1.18,4.89) | 0.0152 |  |
|  |  | Q4 | 159 | 294 | 2.43(1.11,5.34) | 0.0270 |  |
|  | VFA | Q1 | 4 | 452 |  |  |  |
|  |  | Q2 | 17 | 439 | 3.45(1.14,10.47) | 0.0286 |  |
|  |  | Q3 | 77 | 379 | 14.97(5.24,42.75) | <0.0001 |  |
|  |  | Q4 | 186 | 270 | 40.13(13.75,117.09) | <0.0001 |  |
|  | SFA | Q1 | 16 | 440 |  |  |  |
|  |  | Q2 | 48 | 408 | 1.10(0.59,2.07) | 0.7660 |  |
|  |  | Q3 | 73 | 383 | 1.09(0.58,2.06) | 0.7838 |  |
|  |  | Q4 | 147 | 309 | 1.32(0.67,2.62) | 0.4264 |  |
|  | WC | Q1 | 8 | 448 |  |  |  |
|  |  | Q2 | 32 | 424 | 2.72(1.21,6.13) | 0.0153 |  |
|  |  | Q3 | 77 | 379 | 5.78(2.60,12.88) | <0.0001 |  |
|  |  | Q4 | 167 | 289 | 11.13(4.59,27.01) | <0.0001 |  |
|  | SMA | Q1 | 39 | 417 |  |  |  |
|  |  | Q2 | 63 | 393 | 1.09(0.68,1.73) | 0.7273 |  |
|  |  | Q3 | 63 | 393 | 0.83(0.51,1.35) | 0.4546 |  |
|  |  | Q4 | 119 | 337 | 1.18(0.72,1.94) | 0.5022 |  |
|  | L3SMI | Q1 | 38 | 418 |  |  |  |
|  |  | Q2 | 49 | 407 | 0.87(0.53,1.42) | 0.5737 |  |
|  |  | Q3 | 67 | 389 | 1.21(0.75,1.97) | 0.4333 |  |
|  |  | Q4 | 130 | 326 | 1.49(0.90,2.46) | 0.1255 |  |

**Supplemental Table 7. Logistic Regression of Body Composition Indices and MASLD in Hypertension Subgroups Among Men(With vs Without Hyperlipidemia)**

| Group | Exposure |  | MASLD | NO-MASLD | OR(95% CI) | P-value | p for interaction |
| --- | --- | --- | --- | --- | --- | --- | --- |
| Hyperlipidemia | BMI | Q1 | 104 | 633 |  |  | 0.2006 |
|  |  | Q2 | 226 | 506 | 1.61(1.21,2.14) | 0.0011 |  |
|  |  | Q3 | 293 | 441 | 1.66(1.23,2.24) | 0.0009 |  |
|  |  | Q4 | 366 | 369 | 1.34(0.94,1.92) | 0.1031 |  |
|  | VFA | Q1 | 63 | 672 |  |  | 0.0671 |
|  |  | Q2 | 234 | 500 | 4.17(3.03,5.74) | <0.0001 |  |
|  |  | Q3 | 288 | 446 | 5.58(4.00,7.77) | <0.0001 |  |
|  |  | Q4 | 404 | 331 | 9.45(6.59,13.53) | <0.0001 |  |
|  | SFA | Q1 | 115 | 620 |  |  | 0.0256 |
|  |  | Q2 | 234 | 500 | 1.45(1.10,1.90) | 0.0086 |  |
|  |  | Q3 | 265 | 469 | 1.38(1.04,1.84) | 0.0279 |  |
|  |  | Q4 | 375 | 360 | 1.83(1.33,2.52) | 0.0002 |  |
|  | WC | Q1 | 84 | 651 |  |  | 0.0493 |
|  |  | Q2 | 202 | 532 | 2.52(1.86,3.43) | <0.0001 |  |
|  |  | Q3 | 295 | 439 | 4.07(2.92,5.68) | <0.0001 |  |
|  |  | Q4 | 408 | 327 | 6.38(4.22,9.65) | <0.0001 |  |
|  | SMA | Q1 | 162 | 573 |  |  | 0.3505 |
|  |  | Q2 | 212 | 522 | 1.06(0.82,1.36) | 0.6788 |  |
|  |  | Q3 | 249 | 485 | 1.09(0.84,1.42) | 0.5312 |  |
|  |  | Q4 | 366 | 369 | 1.48(1.11,1.97) | 0.0083 |  |
|  | L3SMI | Q1 | 162 | 573 |  |  | 0.0085 |
|  |  | Q2 | 222 | 512 | 1.12(0.86,1.44) | 0.3994 |  |
|  |  | Q3 | 247 | 487 | 1.16(0.89,1.51) | 0.2648 |  |
|  |  | Q4 | 358 | 377 | 1.56(1.16,2.08) | 0.00286 |  |
| No-Hyperlipidemia | BMI | Q1 | 21 | 466 |  |  |  |
|  |  | Q2 | 53 | 420 | 1.54(0.89,2.68) | 0.1245 |  |
|  |  | Q3 | 124 | 360 | 2.86(1.64,4.98) | 0.0002 |  |
|  |  | Q4 | 182 | 294 | 2.64(1.40,5.00) | 0.0028 |  |
|  | VFA | Q1 | 11 | 469 |  |  |  |
|  |  | Q2 | 53 | 427 | 3.86(1.95,7.67) | 0.0001 |  |
|  |  | Q3 | 126 | 354 | 9.63(4.85,19.11) | <0.0001 |  |
|  |  | Q4 | 190 | 290 | 14.40(7.01,29.61) | <0.0001 |  |
|  | SFA | Q1 | 20 | 460 |  |  |  |
|  |  | Q2 | 69 | 411 | 1.90(1.10,3.28) | 0.0205 |  |
|  |  | Q3 | 115 | 365 | 2.44(1.40,4.24) | 0.0016 |  |
|  |  | Q4 | 176 | 304 | 2.90(1.59,5.30) | 0.0005 |  |
|  | WC | Q1 | 15 | 465 |  |  |  |
|  |  | Q2 | 42 | 438 | 2.12(1.13,4.00) | 0.0200 |  |
|  |  | Q3 | 135 | 345 | 6.80(3.61,12.78) | <0.0001 |  |
|  |  | Q4 | 188 | 292 | 7.46(3.59,15.50) | <0.0001 |  |
|  | SMA | Q1 | 49 | 431 |  |  |  |
|  |  | Q2 | 84 | 396 | 1.33(0.88,2.00) | 0.1740 |  |
|  |  | Q3 | 105 | 375 | 1.28(0.85,1.94) | 0.2376 |  |
|  |  | Q4 | 142 | 338 | 1.43(0.92,2.22) | 0.1159 |  |
|  | L3SMI | Q1 | 54 | 426 |  |  |  |
|  |  | Q2 | 65 | 415 | 0.95(0.63,1.45) | 0.8221 |  |
|  |  | Q3 | 116 | 364 | 1.41(0.94,2.11) | 0.0967 |  |
|  |  | Q4 | 145 | 335 | 1.50(0.97,2.33) | 0.0714 |  |

**Supplemental Table 8. Logistic Regression of Body Composition Indices and MASLD in Hypertension Subgroups Among Women(With vs Without Hyperlipidemia)**

| Group | Exposure |  | MASLD | NO-MASLD | OR(95% CI) | P-value | p for interaction |
| --- | --- | --- | --- | --- | --- | --- | --- |
| Hyperlipidemia | BMI | Q1 | 25 | 255 |  |  | 0.0031 |
|  |  | Q2 | 70 | 198 | 2.16(1.26,3.71) | 0.00497 |  |
|  |  | Q3 | 94 | 180 | 2.02(1.14,3.59) | 0.01665 |  |
|  |  | Q4 | 148 | 123 | 3.38(1.76,6.49) | 0.00026 |  |
|  | VFA | Q1 | 10 | 264 |  |  | 0.6770 |
|  |  | Q2 | 56 | 217 | 5.22(2.54,10.70) | <0.0001 |  |
|  |  | Q3 | 104 | 169 | 10.52(5.09,21.75) | <0.0001 |  |
|  |  | Q4 | 167 | 106 | 21.86(10.19,46.89) | <0.0001 |  |
|  | SFA | Q1 | 36 | 238 |  |  | 0.2245 |
|  |  | Q2 | 77 | 196 | 1.24(0.76,2.02) | 0.39610 |  |
|  |  | Q3 | 92 | 181 | 1.10(0.66,1.81) | 0.71861 |  |
|  |  | Q4 | 132 | 141 | 1.17(0.66,2.07) | 0.58342 |  |
|  | WC | Q1 | 21 | 253 |  |  | 0.0482 |
|  |  | Q2 | 63 | 210 | 2.56(1.45,4.53) | 0.00117 |  |
|  |  | Q3 | 105 | 168 | 4.56(2.50,8.31) | <0.0001 |  |
|  |  | Q4 | 148 | 125 | 7.24(3.47,15.14) | <0.0001 |  |
|  | SMA | Q1 | 50 | 224 |  |  | 0.6845 |
|  |  | Q2 | 74 | 199 | 1.17(0.74,1.83) | 0.50245 |  |
|  |  | Q3 | 84 | 189 | 1.26(0.79,2.00) | 0.32662 |  |
|  |  | Q4 | 129 | 144 | 1.41(0.85,2.32) | 0.18320 |  |
|  | L3SMI | Q1 | 52 | 222 |  |  | 0.2921 |
|  |  | Q2 | 70 | 203 | 0.96(0.61,1.51) | 0.86033 |  |
|  |  | Q3 | 81 | 192 | 1.04(0.66,1.66) | 0.86043 |  |
|  |  | Q4 | 134 | 139 | 1.27(0.76,2.11) | 0.36305 |  |
| No-Hyperlipidemia | BMI | Q1 | 16 | 449 |  |  |  |
|  |  | Q2 | 21 | 443 | 0.70(0.35,1.40) | 0.31537 |  |
|  |  | Q3 | 93 | 373 | 2.46(1.33,4.55) | 0.00425 |  |
|  |  | Q4 | 177 | 282 | 2.82(1.39,5.72) | 0.00420 |  |
|  | VFA | Q1 | 3 | 461 |  |  |  |
|  |  | Q2 | 24 | 439 | 6.60(1.95,22.30) | 0.00240 |  |
|  |  | Q3 | 79 | 384 | 20.26(6.16,66.56) | <0.0001 |  |
|  |  | Q4 | 201 | 263 | 55.41(16.49,186.25) | <0.0001 |  |
|  | SFA | Q1 | 15 | 449 |  |  |  |
|  |  | Q2 | 49 | 414 | 1.29(0.69,2.43) | 0.42324 |  |
|  |  | Q3 | 91 | 372 | 1.79(0.96,3.33) | 0.06574 |  |
|  |  | Q4 | 152 | 312 | 1.65(0.84,3.26) | 0.14688 |  |
|  | WC | Q1 | 8 | 456 |  |  |  |
|  |  | Q2 | 32 | 431 | 2.82(1.25,6.32) | 0.01207 |  |
|  |  | Q3 | 86 | 377 | 6.99(3.16,15.46) | <0.0001 |  |
|  |  | Q4 | 181 | 283 | 13.01(5.38,31.41) | <0.0001 |  |
|  | SMA | Q1 | 50 | 414 |  |  |  |
|  |  | Q2 | 58 | 405 | 0.87(0.56,1.35) | 0.53090 |  |
|  |  | Q3 | 79 | 384 | 0.89(0.57,1.38) | 0.59269 |  |
|  |  | Q4 | 120 | 344 | 0.81(0.51,1.30) | 0.38994 |  |
|  | L3SMI | Q1 | 44 | 420 |  |  |  |
|  |  | Q2 | 53 | 410 | 0.99(0.62,1.58) | 0.95429 |  |
|  |  | Q3 | 79 | 384 | 1.32(0.84,2.07) | 0.23231 |  |
|  |  | Q4 | 131 | 333 | 1.26(0.77,2.06) | 3.53756 |  |

**Supplemental Table 9. Logistic Regression of Body Composition Indices and MASLD in Hypertension Subgroups Among Men(With vs Without Diabetes)**

| Group | Exposure |  | MASLD | NO-MASLD | OR(95% CI) | P-value | p for interaction |
| --- | --- | --- | --- | --- | --- | --- | --- |
| Diabetes | BMI | Q1 | 42 | 295 |  |  | 0.8296 |
|  |  | Q2 | 99 | 237 | 1.93(1.26,2.96) | 0.00238 |  |
|  |  | Q3 | 142 | 192 | 2.34(1.51,3.63) | 0.00015 |  |
|  |  | Q4 | 184 | 152 | 2.28(1.36,3.82) | 0.00174 |  |
|  | VFA | Q1 | 30 | 306 |  |  | 0.0715 |
|  |  | Q2 | 101 | 235 | 3.66(2.29,5.82) | <0.0001 |  |
|  |  | Q3 | 140 | 195 | 5.48(3.40,8.86) | <0.0001 |  |
|  |  | Q4 | 196 | 140 | 9.11(5.42,15.33) | <0.0001 |  |
|  | SFA | Q1 | 63 | 273 |  |  | 0.0345 |
|  |  | Q2 | 105 | 231 | 1.10(0.74,1.63) | 0.63041 |  |
|  |  | Q3 | 123 | 212 | 1.00(0.66,1.51) | 0.99174 |  |
|  |  | Q4 | 176 | 160 | 1.19(0.76,1.88) | 0.44816 |  |
|  | WC | Q1 | 36 | 300 |  |  | 0.4225 |
|  |  | Q2 | 91 | 245 | 2.71(1.73,4.26) | <0.0001 |  |
|  |  | Q3 | 147 | 188 | 5.04(3.08,8.23) | <0.0001 |  |
|  |  | Q4 | 193 | 143 | 7.47(4.06,13.74) | <0.0001 |  |
|  | SMA | Q1 | 68 | 268 |  |  | 0.8682 |
|  |  | Q2 | 102 | 234 | 1.22(0.83,1.78) | 0.31270 |  |
|  |  | Q3 | 124 | 211 | 1.28(0.86,1.90) | 0.21772 |  |
|  |  | Q4 | 173 | 163 | 1.58(1.03,2.42) | 0.03547 |  |
|  | L3SMI | Q1 | 71 | 265 |  |  | 0.4418 |
|  |  | Q2 | 110 | 226 | 1.38(0.94,2.02) | 0.09999 |  |
|  |  | Q3 | 125 | 210 | 1.33(0.90,1.96) | 0.15956 |  |
|  |  | Q4 | 161 | 175 | 1.44(0.94,2.22) | 0.09290 |  |
| No-Diabetes | BMI | Q1 | 65 | 814 |  |  |  |
|  |  | Q2 | 175 | 704 | 1.65(1.19,2.31) | 0.00302 |  |
|  |  | Q3 | 289 | 590 | 2.10(1.49,2.97) | <0.0001 |  |
|  |  | Q4 | 373 | 505 | 1.55(1.04,2.31) | 0.03198 |  |
|  | VFA | Q1 | 38 | 841 |  |  |  |
|  |  | Q2 | 147 | 732 | 3.44(2.33,5.08) | <0.0001 |  |
|  |  | Q3 | 314 | 564 | 8.36(5.64,12.40) | <0.0001 |  |
|  |  | Q4 | 403 | 476 | 11.59(7.60,17.66) | <0.0001 |  |
|  | SFA | Q1 | 62 | 817 |  |  |  |
|  |  | Q2 | 196 | 683 | 1.97(1.42,2.73) | <0.0001 |  |
|  |  | Q3 | 254 | 624 | 2.07(1.48,2.91) | <0.0001 |  |
|  |  | Q4 | 390 | 489 | 2.84(1.96,4.12) | <0.0001 |  |
|  | WC | Q1 | 48 | 831 |  |  |  |
|  |  | Q2 | 158 | 721 | 2.72(1.89,3.91) | <0.0001 |  |
|  |  | Q3 | 290 | 588 | 4.87(3.30,7.19) | <0.0001 |  |
|  |  | Q4 | 406 | 473 | 5.83(3.67,9.26) | <0.0001 |  |
|  | SMA | Q1 | 133 | 746 |  |  |  |
|  |  | Q2 | 189 | 690 | 1.13(0.87,1.48) | 0.36090 |  |
|  |  | Q3 | 222 | 656 | 1.08(0.83,1.42) | 0.55853 |  |
|  |  | Q4 | 358 | 521 | 1.50(1.12,2.01) | 0.00627 |  |
|  | L3SMI | Q1 | 135 | 744 |  |  |  |
|  |  | Q2 | 178 | 701 | 1.06(0.81,1.39) | 0.65168 |  |
|  |  | Q3 | 236 | 642 | 1.25(0.95,1.64) | 0.10640 |  |
|  |  | Q4 | 353 | 526 | 1.72(1.28,2.31) | 0.00036 |  |

**Supplemental Table 10. Logistic Regression of Body Composition Indices and MASLD in Hypertension Subgroups Among Women(With vs Without Diabetes)**

| Group | Exposure |  | MASLD | NO-MASLD | OR(95% CI) | P-value | p for interaction |
| --- | --- | --- | --- | --- | --- | --- | --- |
| Diabetes | BMI | Q1 | 15 | 142 |  |  | 0.4487 |
|  |  | Q2 | 38 | 116 | 1.91(0.96,3.82) | 0.06497 |  |
|  |  | Q3 | 64 | 91 | 2.99(1.44,6.21) | 0.00337 |  |
|  |  | Q4 | 90 | 66 | 3.33(1.35,8.23) | 0.00908 |  |
|  | VFA | Q1 | 6 | 150 |  |  | 0.6567 |
|  |  | Q2 | 35 | 120 | 6.13(2.46,15.25) | 0.00010 |  |
|  |  | Q3 | 72 | 83 | 15.04(6.01,37.62) | <0.0001 |  |
|  |  | Q4 | 94 | 62 | 19.74(7.46,52.22) | <0.0001 |  |
|  | SFA | Q1 | 23 | 133 |  |  | 0.1131 |
|  |  | Q2 | 48 | 107 | 1.27(0.69,2.33) | 0.44763 |  |
|  |  | Q3 | 55 | 100 | 1.06(0.55,2.02) | 0.86378 |  |
|  |  | Q4 | 81 | 75 | 1.08(0.51,2.26) | 0.84512 |  |
|  | WC | Q1 | 14 | 142 |  |  | 0.0598 |
|  |  | Q2 | 43 | 112 | 2.65(1.32,5.35) | 0.00633 |  |
|  |  | Q3 | 64 | 91 | 4.14(1.95,8.79) | 0.00022 |  |
|  |  | Q4 | 86 | 70 | 4.50(1.75,11.59) | 0.00184 |  |
|  | SMA | Q1 | 35 | 121 |  |  | 0.8191 |
|  |  | Q2 | 45 | 110 | 1.14(0.65,2.01) | 0.65069 |  |
|  |  | Q3 | 48 | 107 | 1.04(0.58,1.84) | 0.90618 |  |
|  |  | Q4 | 79 | 77 | 1.15(0.61,2.17) | 0.65732 |  |
|  | L3SMI | Q1 | 35 | 121 |  |  | 0.9969 |
|  |  | Q2 | 36 | 119 | 0.84(0.47,1.50) | 0.55605 |  |
|  |  | Q3 | 57 | 98 | 1.46(0.83,2.57) | 0.18665 |  |
|  |  | Q4 | 79 | 77 | 1.12(0.59,2.12) | 0.72351 |  |
| No-Diabetes | BMI | Q1 | 23 | 563 |  |  |  |
|  |  | Q2 | 44 | 533 | 1.10(0.63,1.90) | 0.74338 |  |
|  |  | Q3 | 135 | 446 | 2.43(1.44,4.08) | 0.00082 |  |
|  |  | Q4 | 235 | 346 | 2.80(1.57,5.01) | 0.00052 |  |
|  | VFA | Q1 | 7 | 575 |  |  |  |
|  |  | Q2 | 33 | 548 | 3.77(1.63,8.73) | 0.00189 |  |
|  |  | Q3 | 127 | 454 | 14.05(6.27,31.47) | <0.0001 |  |
|  |  | Q4 | 270 | 311 | 32.81(14.32,75.17) | <0.0001 |  |
|  | SFA | Q1 | 28 | 554 |  |  |  |
|  |  | Q2 | 70 | 511 | 0.98(0.60,1.61) | 0.93549 |  |
|  |  | Q3 | 131 | 450 | 1.42(0.87,2.30) | 0.15617 |  |
|  |  | Q4 | 208 | 373 | 1.36(0.80,2.31) | 0.26076 |  |
|  | WC | Q1 | 16 | 566 |  |  |  |
|  |  | Q2 | 42 | 539 | 1.96(1.07,3.62) | 0.03034 |  |
|  |  | Q3 | 130 | 451 | 5.98(3.29,10.85) | <0.0001 |  |
|  |  | Q4 | 249 | 332 | 11.97(6.04,23.73) | <0.0001 |  |
|  | SMA | Q1 | 63 | 519 |  |  |  |
|  |  | Q2 | 88 | 493 | 0.96(0.66,1.41) | 0.84048 |  |
|  |  | Q3 | 109 | 472 | 0.98(0.66,1.45) | 0.92039 |  |
|  |  | Q4 | 177 | 404 | 1.09(0.72,1.63) | 0.69380 |  |
|  | L3SMI | Q1 | 64 | 518 |  |  |  |
|  |  | Q2 | 79 | 502 | 0.90(0.61,1.33) | 0.60187 |  |
|  |  | Q3 | 110 | 471 | 1.11(0.76,1.64) | 0.58399 |  |
|  |  | Q4 | 184 | 397 | 1.13(0.74,1.72) | 0.56163 |  |

**Supplemental Table 11. Logistic Regression of Body Composition Indices and MASLD in Hypertension Subgroups Among Men(With vs Without Hyperuricemia)**

| Group | Exposure |  | MASLD | NO-MASLD | OR(95% CI) | P-value | p for interaction |
| --- | --- | --- | --- | --- | --- | --- | --- |
| Hyperuricemia | BMI | Q1 | 50 | 780 |  |  | 0.0063 |
|  |  | Q2 | 149 | 677 | 1.60(1.11,2.31) | 0.01161 |  |
|  |  | Q3 | 250 | 577 | 1.31(0.89,1.92) | 0.17323 |  |
|  |  | Q4 | 359 | 469 | 0.89(0.56,1.42) | 0.62450 |  |
|  | VFA | Q1 | 34 | 794 |  |  | 0.1020 |
|  |  | Q2 | 109 | 719 | 2.81(1.91,4.12) | <0.0001 |  |
|  |  | Q3 | 280 | 547 | 3.40(2.29,5.05) | <0.0001 |  |
|  |  | Q4 | 385 | 443 | 5.80(3.75,8.97) | <0.0001 |  |
|  | SFA | Q1 | 63 | 765 |  |  | 0.3404 |
|  |  | Q2 | 165 | 663 | 1.95(1.36,2.80) | 0.00026 |  |
|  |  | Q3 | 227 | 600 | 1.73(1.18,2.53) | 0.00502 |  |
|  |  | Q4 | 353 | 475 | 2.42(1.57,3.71) | <0.0001 |  |
|  | WC | Q1 | 42 | 786 |  |  | 0.1227 |
|  |  | Q2 | 121 | 707 | 2.57(1.73,3.81) | <0.0001 |  |
|  |  | Q3 | 264 | 563 | 3.86(2.49,5.98) | <0.0001 |  |
|  |  | Q4 | 381 | 447 | 3.93(2.25,6.86) | <0.0001 |  |
|  | SMA | Q1 | 119 | 709 |  |  | 0.1240 |
|  |  | Q2 | 168 | 660 | 1.13(0.81,1.59) | 0.46971 |  |
|  |  | Q3 | 206 | 621 | 1.10(0.77,1.57) | 0.61591 |  |
|  |  | Q4 | 315 | 513 | 1.58(1.07,2.33) | 0.02222 |  |
|  | L3SMI | Q1 | 125 | 703 |  |  | 0.2399 |
|  |  | Q2 | 166 | 662 | 1.19(0.85,1.67) | 0.31187 |  |
|  |  | Q3 | 209 | 618 | 1.34(0.94,1.90) | 0.10478 |  |
|  |  | Q4 | 308 | 520 | 1.62(1.10,2.38) | 0.01532 |  |
| No-Hyperuricemia | BMI | Q1 | 71 | 316 |  |  |  |
|  |  | Q2 | 147 | 240 | 2.00(1.39,2.88) | 0.00021 |  |
|  |  | Q3 | 156 | 230 | 2.42(1.66,3.53) | <0.0001 |  |
|  |  | Q4 | 187 | 200 | 2.22(1.44,3.42) | 0.00028 |  |
|  | VFA | Q1 | 54 | 333 |  |  |  |
|  |  | Q2 | 136 | 251 | 2.79(1.84,4.24) | <0.0001 |  |
|  |  | Q3 | 155 | 231 | 8.07(5.35,12.18) | <0.0001 |  |
|  |  | Q4 | 216 | 171 | 11.76(7.56,18.28) | <0.0001 |  |
|  | SFA | Q1 | 67 | 320 |  |  |  |
|  |  | Q2 | 146 | 241 | 1.35(0.97,1.88) | 0.07927 |  |
|  |  | Q3 | 149 | 237 | 1.42(1.01,2.00) | 0.04466 |  |
|  |  | Q4 | 199 | 188 | 1.74(1.20,2.53) | 0.00377 |  |
|  | WC | Q1 | 53 | 334 |  |  |  |
|  |  | Q2 | 128 | 259 | 2.36(1.60,3.49) | <0.0001 |  |
|  |  | Q3 | 175 | 211 | 5.17(3.45,7.74) | <0.0001 |  |
|  |  | Q4 | 205 | 182 | 6.68(4.16,10.73) | <0.0001 |  |
|  | SMA | Q1 | 96 | 291 |  |  |  |
|  |  | Q2 | 124 | 263 | 1.05(0.80,1.40) | 0.70931 |  |
|  |  | Q3 | 141 | 245 | 1.08(0.81,1.43) | 0.61671 |  |
|  |  | Q4 | 200 | 187 | 1.40(1.04,1.90) | 0.02854 |  |
|  | L3SMI | Q1 | 94 | 293 |  |  |  |
|  |  | Q2 | 125 | 262 | 1.06(0.80,1.40) | 0.67728 |  |
|  |  | Q3 | 151 | 235 | 1.13(0.85,1.49) | 0.41118 |  |
|  |  | Q4 | 191 | 196 | 1.45(1.07,1.97) | 0.01621 |  |

**Supplemental Table 12. Logistic Regression of Body Composition Indices and MASLD in Hypertension Subgroups Among Women(With vs Without Hyperuricemia)**

| Group | Exposure |  | MASLD | NO-MASLD | OR(95% CI) | P-value | p for interaction |
| --- | --- | --- | --- | --- | --- | --- | --- |
| Hyperuricemia | BMI | Q1 | 8 | 59 |  |  | 0.4948 |
|  |  | Q2 | 22 | 44 | 2.70(1.00,7.27) | 0.04891 |  |
|  |  | Q3 | 25 | 41 | 2.69(0.92,7.83) | 0.06937 |  |
|  |  | Q4 | 45 | 22 | 8.86(2.28,34.48) | 0.00164 |  |
|  | VFA | Q1 | 5 | 62 |  |  | 0.2921 |
|  |  | Q2 | 21 | 45 | 4.56(1.51,13.75) | 0.00710 |  |
|  |  | Q3 | 31 | 35 | 6.35(1.98,20.37) | 0.00188 |  |
|  |  | Q4 | 43 | 24 | 12.23(3.36,44.52) | 0.00015 |  |
|  | SFA | Q1 | 14 | 53 |  |  | 0.2745 |
|  |  | Q2 | 22 | 44 | 0.82(0.34,1.98) | 0.66228 |  |
|  |  | Q3 | 29 | 37 | 0.84(0.32,2.17) | 0.71593 |  |
|  |  | Q4 | 35 | 32 | 0.62(0.20,1.86) | 0.39095 |  |
|  | WC | Q1 | 7 | 60 |  |  | 0.1071 |
|  |  | Q2 | 23 | 43 | 3.61(1.29,10.07) | 0.01426 |  |
|  |  | Q3 | 28 | 38 | 4.91(1.54,15.66) | 0.00708 |  |
|  |  | Q4 | 42 | 25 | 11.13(2.39,51.86) | 0.00214 |  |
|  | SMA | Q1 | 14 | 53 |  |  | 0.6630 |
|  |  | Q2 | 24 | 42 | 1.21(0.52,2.85) | 0.65933 |  |
|  |  | Q3 | 26 | 40 | 1.18(0.49,2.83) | 0.71462 |  |
|  |  | Q4 | 36 | 31 | 1.20(0.45,3.23) | 0.71230 |  |
|  | L3SMI | Q1 | 15 | 52 |  |  | 0.2576 |
|  |  | Q2 | 22 | 44 | 1.15(0.49,2.71) | 0.74855 |  |
|  |  | Q3 | 30 | 36 | 1.37(0.57,3.28) | 0.48353 |  |
|  |  | Q4 | 33 | 34 | 0.76(0.28,2.07) | 0.58829 |  |
| No-Hyperuricemia | BMI | Q1 | 24 | 647 |  |  |  |
|  |  | Q2 | 69 | 601 | 1.51(0.92,2.50) | 0.10614 |  |
|  |  | Q3 | 168 | 502 | 2.75(1.67,4.51) | <0.0001 |  |
|  |  | Q4 | 283 | 387 | 2.88(1.63,5.07) | 0.00027 |  |
|  | VFA | Q1 | 9 | 662 |  |  |  |
|  |  | Q2 | 45 | 625 | 4.12(1.98,8.61) | 0.00016 |  |
|  |  | Q3 | 161 | 509 | 14.59(7.17,29.71) | <0.0001 |  |
|  |  | Q4 | 329 | 341 | 34.07(16.38,70.88) | <0.0001 |  |
|  | SFA | Q1 | 36 | 635 |  |  |  |
|  |  | Q2 | 99 | 571 | 1.16(0.75,1.79) | 0.49489 |  |
|  |  | Q3 | 161 | 509 | 1.45(0.94,2.22) | 0.09070 |  |
|  |  | Q4 | 248 | 422 | 1.33(0.83,2.14) | 0.23991 |  |
|  | WC | Q1 | 18 | 653 |  |  |  |
|  |  | Q2 | 68 | 602 | 2.84(1.63,4.94) | 0.00022 |  |
|  |  | Q3 | 161 | 509 | 6.52(3.75,11.36) | <0.0001 |  |
|  |  | Q4 | 297 | 373 | 12.26(6.49,23.15) | <0.0001 |  |
|  | SMA | Q1 | 87 | 584 |  |  |  |
|  |  | Q2 | 114 | 556 | 1.00(0.71,1.40) | 0.98964 |  |
|  |  | Q3 | 127 | 543 | 0.87(0.62,1.24) | 0.44692 |  |
|  |  | Q4 | 216 | 454 | 1.08(0.75,1.55) | 0.69416 |  |
|  | L3SMI | Q1 | 84 | 587 |  |  |  |
|  |  | Q2 | 101 | 569 | 0.93(0.66,1.32) | 0.68520 |  |
|  |  | Q3 | 128 | 542 | 1.08(0.76,1.52) | 0.67061 |  |
|  |  | Q4 | 231 | 439 | 1.29(0.89,1.86) | 0.18527 |  |

**Supplemental Table 13. Sex-Stratified Propensity Score Matching Results (Model 3)**

| Variable | | Men | | | | Women | | | |
| --- | --- | --- | --- | --- | --- | --- | --- | --- | --- |
|  |  | Case | Control |  |  | Case | Control |  |  |
|  |  | 1396 | 2738 | OR (95% CI) | P-value | 644 | 1288 | OR (95% CI) | P-value |
| BMI | Q1 |  |  | 1(reference) |  |  |  | 1(reference) |  |
|  | Q2 |  |  | 2.06(1.65,2.59) | <0.0001 |  |  | 2.50(1.79,3.49) | <0.0001 |
|  | Q3 |  |  | 3.26(2.59,4.09) | <0.0001 |  |  | 3.45(2.45,4.85) | <0.0001 |
|  | Q4 |  |  | 5.97(4.69,7.61) | <0.0001 |  |  | 7.26(5.09,10.34) | <0.0001 |
| VFA | Q1 |  |  | 1(reference) |  |  |  | 1(reference) |  |
|  | Q2 |  |  | 4.29(3.29,5.59) | <0.0001 |  |  | 5.32(3.37,8.40) | <0.0001 |
|  | Q3 |  |  | 6.99(5.32,9.18) | <0.0001 |  |  | 14.22(8.84,22.88) | <0.0001 |
|  | Q4 |  |  | 14.27(10.77,18.90) | <0.0001 |  |  | 35.54(21.59,58.50) | <0.0001 |
| SFA | Q1 |  |  | 1(reference) |  |  |  | 1(reference) |  |
|  | Q2 |  |  | 1.74(1.41,2.15) | <0.0001 |  |  | 1.06(0.79,1.41) | 0.70665 |
|  | Q3 |  |  | 2.38(1.92,2.95) | <0.0001 |  |  | 1.64(1.24,2.18) | 0.00054 |
|  | Q4 |  |  | 4.27(3.43,5.33) | <0.0001 |  |  | 2.00(1.51,2.68) | <0.0001 |
| WC | Q1 |  |  | 1(reference) |  |  |  | 1(reference) |  |
|  | Q2 |  |  | 4.39(3.33,5.80) | <0.0001 |  |  | 2.76(1.95,3.92) | <0.0001 |
|  | Q3 |  |  | 9.43(6.99,12.74) | <0.0001 |  |  | 5.10(3.51,7.43) | <0.0001 |
|  | Q4 |  |  | 28.68(20.43,40.26) | <0.0001 |  |  | 13.48(8.93,20.35) | <0.0001 |
| SMA | Q1 |  |  | 1(reference) |  |  |  | 1(reference) |  |
|  | Q2 |  |  | 1.27(1.04,1.56) | 0.0197 |  |  | 1.22(0.92,1.62) | 0.16050 |
|  | Q3 |  |  | 1.54(1.27,1.87) | <0.0001 |  |  | 1.44(1.09,1.91) | 0.01120 |
|  | Q4 |  |  | 2.89(2.37,3.52) | <0.0001 |  |  | 2.29(1.73,3.04) | <0.0001 |
| L3SMI | Q1 |  |  | 1(reference) |  |  |  | 1(reference) |  |
|  | Q2 |  |  | 1.18(0.97,1.45) | 0.0957 |  |  | 1.05(0.80,1.40) | 0.71004 |
|  | Q3 |  |  | 1.56(1.28,1.90) | <0.0001 |  |  | 1.57(1.19,2.07) | 0.00131 |
|  | Q4 |  |  | 2.50(2.05,3.05) | <0.0001 |  |  | 2.17(1.64,2.88) | <0.0001 |


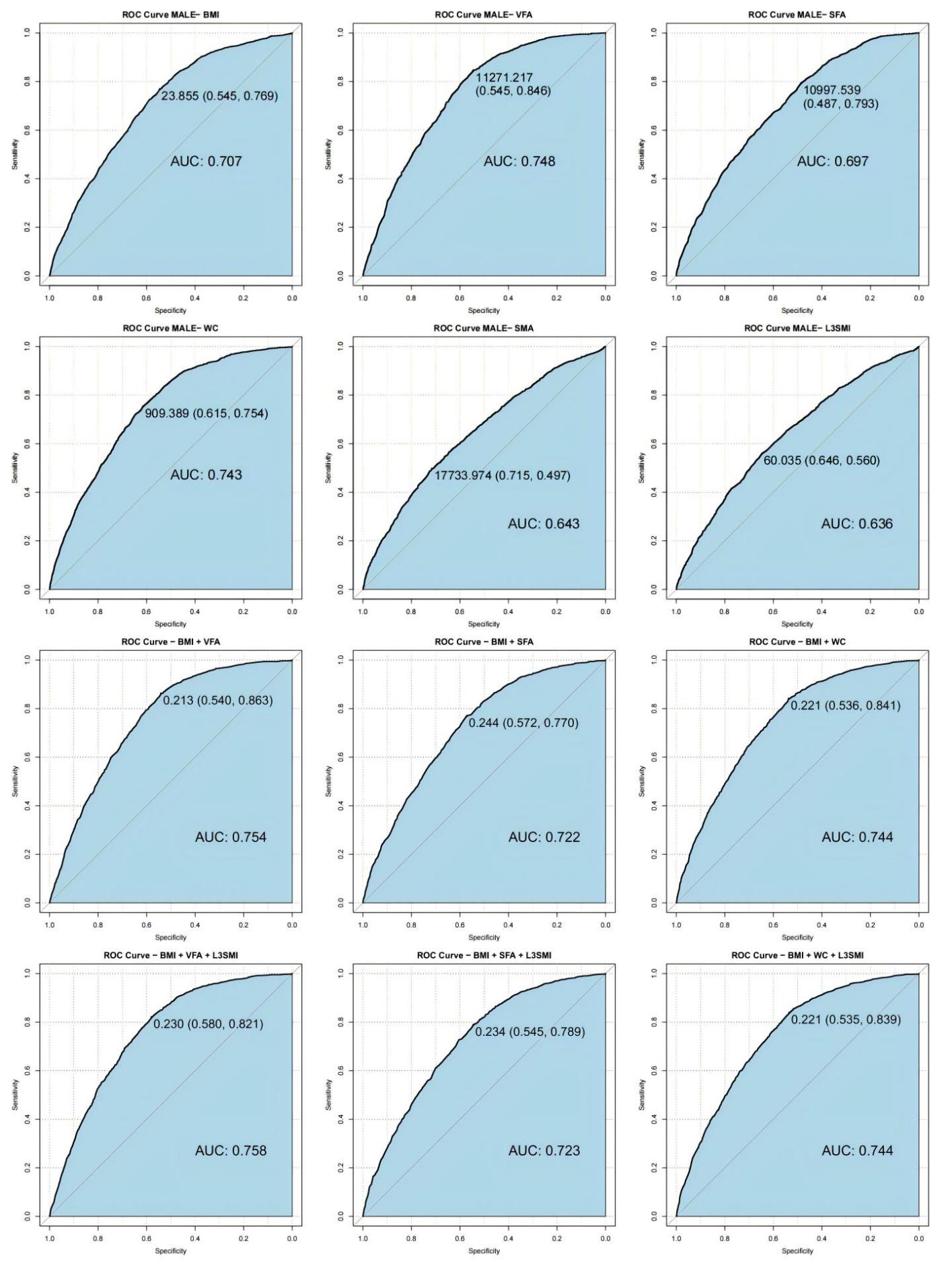


**Supplemental Figure 1. ROC Curves Comparing Predictive Performance of VFA, SFA, BMI, WC,SMA and L3SMI for MASLD in Men.**


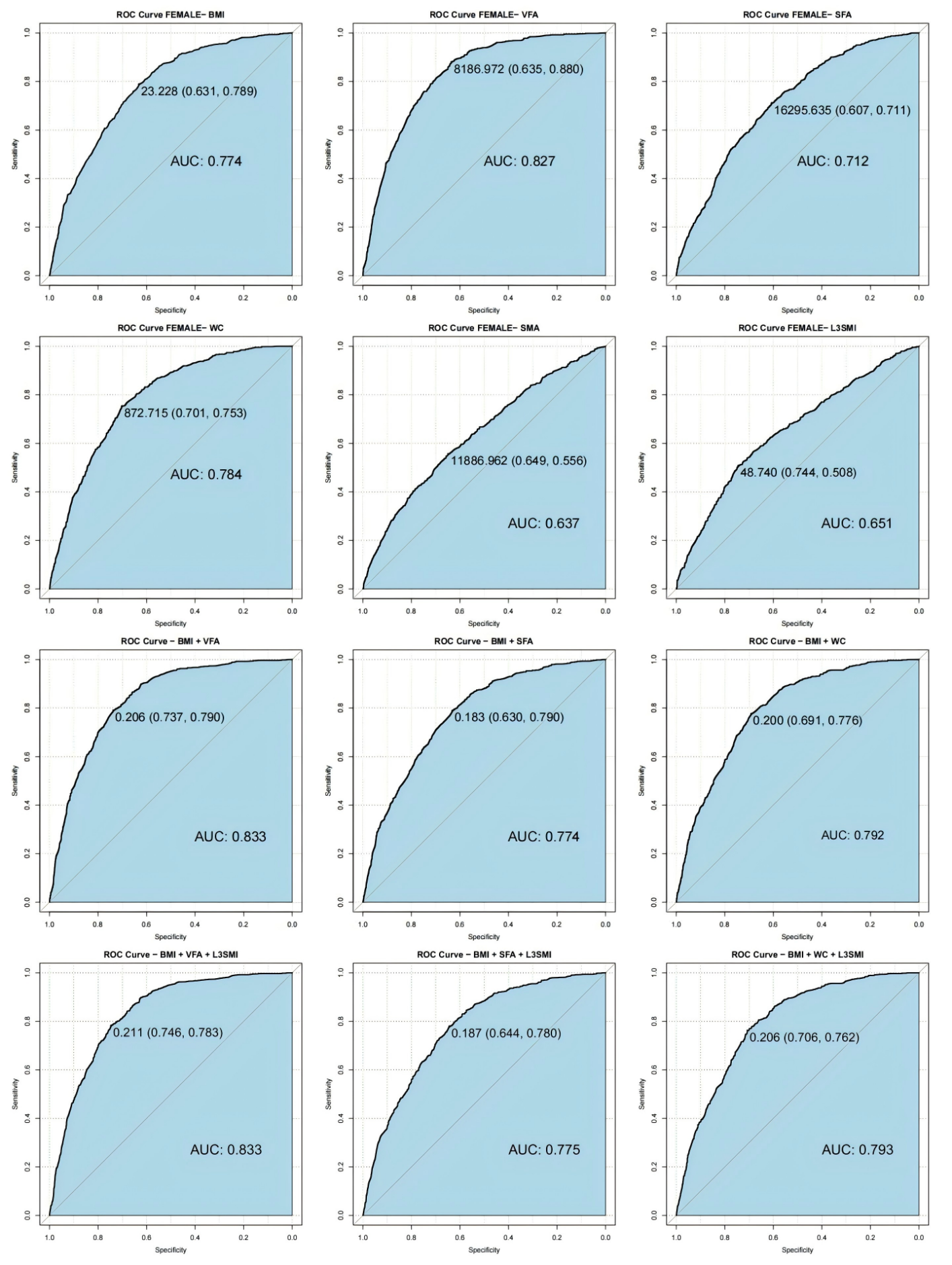


**Supplemental Figure 2.** ROC Curves Comparing Predictive Performance of VFA, SFA, BMI, WC,SMA and L3SMI for MASLD in Women.
